# SERS Immunosensors for Cancer Markers Detection

**DOI:** 10.3390/ma16103733

**Published:** 2023-05-15

**Authors:** Georgia Geka, Anastasia Kanioura, Vlassis Likodimos, Spiros Gardelis, Nikolaos Papanikolaou, Sotirios Kakabakos, Panagiota Petrou

**Affiliations:** 1 Immunoassays/Immunosensors Lab, Institute of Nuclear & Radiological Sciences & Technology, Energy & Safety, NCSR “Demokritos”, 15341 Aghia Paraskevi, Greece; georgia.geka101@gmail.com (G.G.); nkanioura@ipta.demokritos.gr (A.K.); skakab@rrp.demokritos.gr (S.K.); 2 Section of Condensed Matter Physics, Department of Physics, National and Kapodistrian University of Athens, University Campus, 15784 Athens, Greece; vlikodimos@phys.uoa.gr (V.L.); sgardelis@phys.uoa.gr (S.G.); 3 Institute of Nanoscience & Nanotechnology, NCSR “Demokritos”, 15341 Aghia Paraskevi, Greece; n.papanikolaou@inn.demokritos.gr

**Keywords:** Raman spectroscopy, nanomaterials, cancer markers

## Abstract

Early diagnosis and monitoring are essential for the effective treatment and survival of patients with different types of malignancy. To this end, the accurate and sensitive determination of substances in human biological fluids related to cancer diagnosis and/or prognosis, i.e., cancer biomarkers, is of ultimate importance. Advancements in the field of immunodetection and nanomaterials have enabled the application of new transduction approaches for the sensitive detection of single or multiple cancer biomarkers in biological fluids. Immunosensors based on surface-enhanced Raman spectroscopy (SERS) are examples where the special properties of nanostructured materials and immunoreagents are combined to develop analytical tools that hold promise for point-of-care applications. In this frame, the subject of this review article is to present the advancements made so far regarding the immunochemical determination of cancer biomarkers by SERS. Thus, after a short introduction about the principles of both immunoassays and SERS, an extended presentation of up-to-date works regarding both single and multi-analyte determination of cancer biomarkers is presented. Finally, future perspectives on the field of SERS immunosensors for cancer markers detection are briefly discussed.

## 1. Introduction

The ability to detect disease biomarkers in human body fluids such as blood, urine, and saliva with high sensitivity and specificity is crucial for both the early diagnosis and successful treatment of diseases. To this end, immunochemical methods based on antigen-antibody interactions have been established in everyday clinical practice as the methods of choice for the sensitive and reliable detection of protein-based cancer biomarkers in bodily fluids [1]. In addition to their excellent analytical performance (high sensitivity and specificity and relatively short turnaround times), the widespread use of immunochemical methods for cancer biomarkers detection is also due to their incorporation into fully automated high-throughput analyzers that are well suited to the heavy workflow of hospital clinical laboratories. On the other hand, there are solutions with less complexity and size that are more suitable for use in emergency departments or close to patients in intensive care units. Therefore, immunochemical methods have monopolized the field of protein biomarker diagnostics with respect to other analytical techniques, such as mass spectroscopy. For other types of cancer markers, such as microRNA or circulating tumor cells, other detection methods, such as molecular (e.g., real-time PCR) and cell sorting techniques (e.g., flow cytometry), respectively, are the most widely employed [1].

Various types of immunochemical methods have been developed over the years, using different types of labels such as enzymes in combination with chromogenic or luminescent substrates, fluorescent compounds organic or inorganic, noble metal nanoparticles or nanoparticles of organic or inorganic nature, etc. Despite the diversity of labels and assay formats, there are some limitations regarding the application of standard immunochemical techniques outside the laboratory. To fill this gap, over recent decades, immunosensors have been developed aiming to combine the analytical performance of immunochemical methods with portability and thus move the analysis from the lab to the field or the patient’s side [1,2].

**Figure 1:**
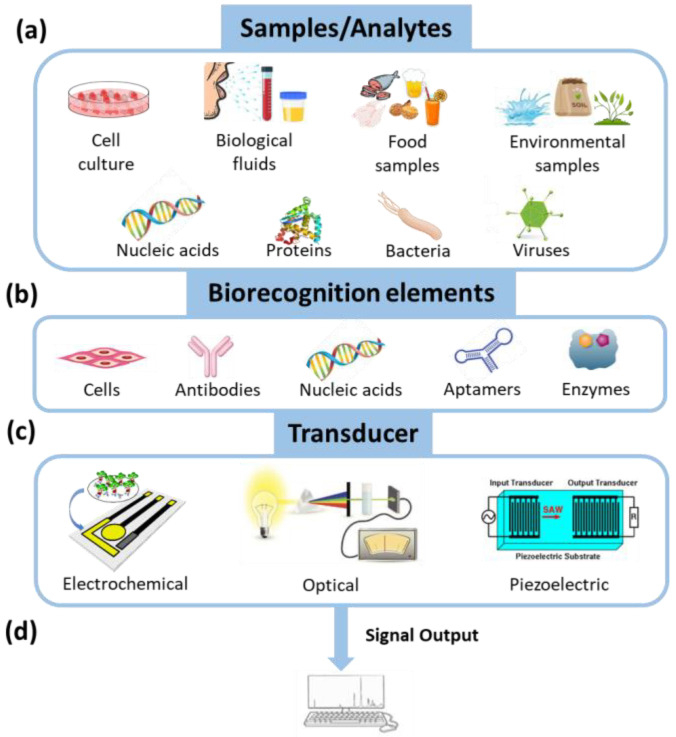
Basic components of a biosensor: (**a**) the analyte: the substance of interest which is needed to be detected in the sample; (**b**) the biorecognition element: a molecule that recognizes specifically the analyte; (**c**) the transducer: converts the analyte-biorecognition element binding into a measurable signal; (**d**) the signal processing and display: a combination of hardware and software to transform the transducer response to the analyte concentration in the sample.

Immunosensors are a subcategory of biosensors (Figure 1) in which the biorecognition element is an antibody or an antigen [1,2,3,4,5,6,7,8,9]. Thus, compared to other types of biosensors, such as DNA, enzymatic, or cell sensors, they inherit all the advantages of standard immunochemical methods, such as the high sensitivity and specificity, the reliability of the determinations, and the ability to detect the analyte of interest in complex media with the potential for multiplexed analysis and on-site use. Although antibody-based biosensors appeared in the literature for the first time in the 1950s, the field greatly expanded after the mid-1970s [1,2]. Over the years, additional features have been incorporated into the developed immunosensors, such as the ability for multiplexed determination of different biomarkers in a single run, the label-free real-time monitoring of binding reactions, the automatic performance of the assay steps, and the considerable miniaturization of transducers and related instruments. Furthermore, the implementation of nanomaterials in both immunoassays and immunosensors, either through their incorporation on the transducer surface or as labels, considerably improved the detection sensitivity as they provided for high surface area for the attachment of immunoreagents, thus facilitating the analyte’s access to the immobilized antibodies and/or lead to signal amplification [10].

Regarding the transducer part, immunosensing has been combined with both optical [3,4,5,6,7] and electrochemical transducers [3,8,9] and, to a lesser extent, with other types of transducers to develop assays for the detection of specific analytes in different matrices. Optical detection is considered more appropriate for multiplexed analyte detection in complex matrices, compared to electrochemical detection, since the signal is less affected by matrix interferences and cross-talk effects between adjacent immunosensors [5,6]. Moreover, there is a great variety of available transduction principles, including colorimetry and photoluminescence (mainly fluorescence) [3,4], reflectance spectroscopy [11], surface plasmon resonance (SPR) [5], interferometry [5], and surface-enhanced Raman spectroscopy (SERS) [12].

In particular, SERS-based immunosensors have been successfully employed in studies related to in vitro diagnostics [13], environmental monitoring [14], and food quality and safety assessment [15]. In all these applications, the antigen-antibody interaction contributes to the high specificity of the assays developed, while detection by SERS provides high sensitivity that can reach the single molecule level [13]. To this end, the implementation of appropriate SERS substrates as solid carriers for immunochemical determination of analytes is critical since it is one of the main factors contributing to increased detection sensitivity of SERS-based immunosensors as compared to the standard immunochemical techniques, e.g., enzyme-linked immunosorbent assays (ELISAs) as well as to other types of optical immunosensors [10,14,16]. An additional advantage of SERS immunosensors compared to other optical transduction principles is the relatively low cost of SERS substrates (as compared, for example, to SPR chips) and the ease of their fabrication that does not require clean room facilities (as compared, for example, to silicon-based interferometric sensor chips). In this frame, the aim of this review is to present the advances in the development of SERS-based immunosensors, especially those applied for the detection of cancer biomarkers. At first, the principle of SERS, the substrates, and Raman tags so far implemented in immunosensing will be presented, followed by a detailed discussion of SERS applications in the field of cancer biomarker detection. Finally, the challenges and future aspects in this application field will be commented on.

## 2. Surface-Enhanced Raman Spectroscopy (SERS) 

**Figure 2:**
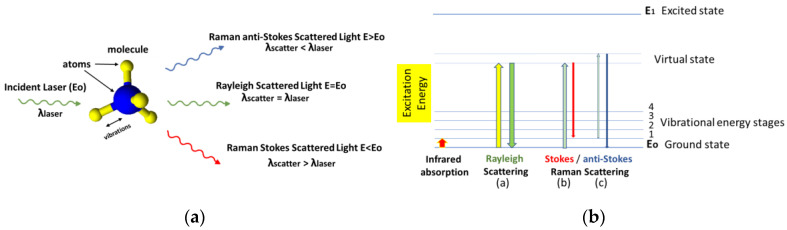
Principle of Raman scattering. (**a**) Raman and Rayleigh scattering. (**b**) Schematic diagram of the energy transitions involved in Rayleigh scattering and Raman scattering. Raman scattering occurs through the interaction of an incident photon with a molecular vibration mode that leads to gaining (anti-Stokes scattering, blue-shifted) or losing (Stokes scattering, red-shifted) an amount of energy corresponding to that vibrational mode.

Raman spectroscopy is an analytical technique where scattered light is used to measure the vibrational energy modes of a molecule. It has been named after the Indian physicist C.V. Raman who, together with his research partner K.S. Krishnan, were the first to observe Raman scattering in 1928. Raman spectroscopy can be used for the identification of substances through their characteristic Raman «fingerprint» [17,18]. When electromagnetic radiation is illuminated on a molecule, the radiation interacts with the electrons, and the result is Rayleigh elastic scattering and a much weaker inelastic scattering event called Raman scattering. In Raman scattering, the scattered light is of a different wavelength from the irradiated (Figure 2a). More specifically, the incident light photon can lose energy by exciting a vibrational mode in a molecule, and the wavelength of the scattered photon becomes larger (Stokes), or the incident photon strikes the molecule, which can already be at an excited vibrational state and the scattered photon shifts to a shorter wavelength (anti-Stokes) as the molecule goes to its ground vibrational state. It should be noted that Raman scattering differs from fluorescence, wherein the incident light is entirely absorbed by the molecule, which is transferred to an excited electronic state from where it goes to a lower electronic state after a certain period by emission of a photon. The Raman shift does not depend on the frequency of incident light, but the intensity of the signal can be affected by the choice of excitation wavelength (Figure 2b).

Due to the mechanism involved, Raman spectroscopy allows for the accurate identification of organic, inorganic, and biological species, an advantage that lack many other analytical techniques, such as ultraviolet absorbance and fluorescence spectroscopy [19]. In addition, Raman spectroscopy requires minimal sample treatment prior to analysis and therefore is well-fitted for on-site determination, especially since portable instruments have become available. However, the weak intensity of the Raman spectrum, which is attributed to rare inelastic scattering, limits its application to fields where the concentration of the analyte of interest is very low or the matrix is very complex. For this reason, metal substrates made of Au, Ag, Cu, and other metals have been employed to enhance the local electromagnetic field and increase the efficiency of Raman scattering by several orders of magnitude, leading to surface-enhanced Raman spectroscopy. Thus, surface-enhanced Raman spectroscopy (or SERS) bridges Raman spectroscopy and materials science enabling the non-destructive, ultrasensitive, and selective detection of analytes of both low [20,21,22,23] and high molecular weight [24,25], as well as of whole cells both in vitro and in vivo [26,27,28,29].

**Figure 3:**
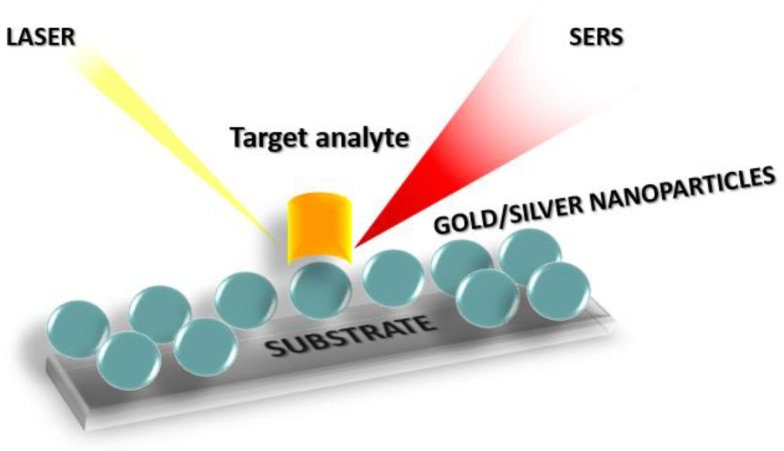
Illustration of the SERS effect: Raman scattering, intrinsically weak, is strongly amplified if the molecules are placed at the surface of a nanostructured metallic substrate.

Two different mechanisms participate in the SERS effect, i.e., the Raman signal enhancement from nanostructured noble metals, the electromagnetic mechanism, and the chemical mechanism [20]. The electromagnetic mechanism, which is the dominant SERS enhancement mechanism, is related to the excitation of plasmons in the metal nanostructures by the incident light that results locally in an enhanced electromagnetic field which in turn is responsible for the large enhancement in Raman scattering. The chemical mechanism, on the other hand, depends strongly on the nature of the molecule and involves the formation of new molecular states and chemical bonds due to the direct interaction of the molecules with the SERS surface. In this mechanism, the molecular polarizability tensor is significantly enhanced due to a photo-induced charge transfer process between the adsorbed molecules and the molecular monolayer of the substrate [30]. Thus, molecules immobilized onto a SERS substrate could be, in principle, directly detected (Figure 3). However, when an immunochemical technique is employed for the detection of an analyte of interest, the detection can be label-free or employ detection antibodies or antigens labeled with molecules known as Raman labels or tags. Thus, the most widely used SERS substrates and Raman tags are discussed in the next sections.

### 2.1. SERS Substrates

**Figure 4:**
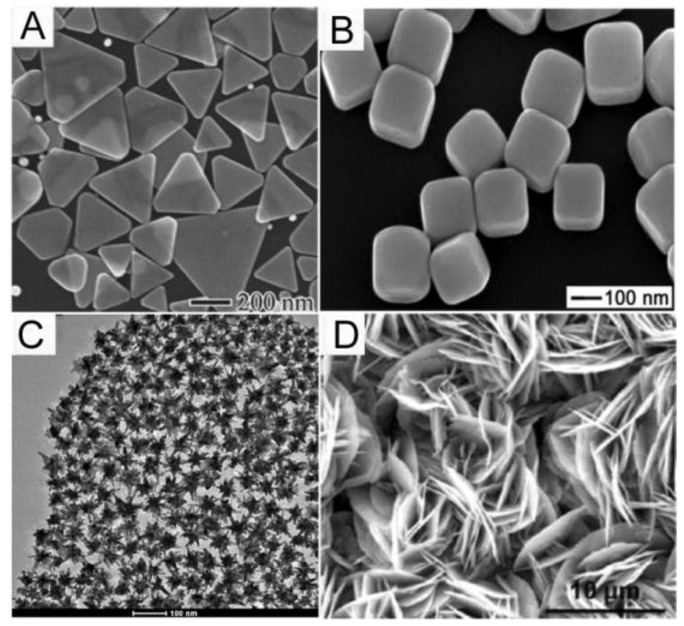
Images of nanostructures with various morphologies for SERS-based detection: (**A**) SEM image of Ag nanoprisms; (**B**) SEM image of Ag nanocubes; (**C**) TEM image of Au nanostars; and (**D**) SEM image of Ag nanosheets [39]. Copyright 2015. Reproduced with permission from MDPI.

The performance of a SERS substrate depends both on the metal used and the metal layer nanostructuring. Generally, gold (Au) and silver (Ag) are the most common metals used for SERS applications [23]. Amongst these two metals, Au has the advantage of being inert towards oxidation and, in general, more biocompatible than Ag [31]. Furthermore, Au nanoparticles present a strong surface plasmon resonance phenomenon when illuminated with light in the visible and near-infrared wavelength ranges (approx. 400–1000 nm) [32]. The resonance of Au surface plasmon upon illumination leads to strong Raman signal enhancement (10–14 orders of magnitude compared to conventional Raman spectroscopy) due to the creation of “hot spots” on aggregated Au particle clusters [32]. Apart from their plasmonic properties, Au and Ag nanoparticles are the most widely exploited SERS substrates for biomolecule detection due to the large surface-to-volume ratio they offer, which enables the immobilization of high quantities of binding molecules to a low volume of nanoparticles, thus, accelerating the immunochemical reactions with respect to planar substrates. Another advantage of nanoparticles is the ease of their fabrication as well as the fact that they can be manipulated by changing the reactant concentration, the temperature and the ionic strength of the solution, enabling control of their size, shape, aggregation properties, and surface charge properties [33]. The most extensively-employed method for the preparation of both Au and Ag nanoparticles relies on sodium citrate reduction of HAuC1_4_ and AgNO_3_ solutions, respectively [34]. Nonetheless, up to date, various modifications of these methods for synthesizing SERS active substrates in the form of metal nanoparticles with different morphologies, such as nanorods [35], nanospheres [36], nanocubes [37], and nanocages [38], have been developed. Several studies have shown that the size and shape of the nanoparticles affect the SERS enhancement achieved. For example, it has been reported that SERS enhancement is more intense when the nanoparticles are rod-shaped compared to spherical [32]. It has also been shown that considerable enhancement of the Raman signal can be achieved by increasing the sharpness and roughness of metal nanoparticles due to plasmonic “hot spot” localization at the sharp edges and nanogaps of these nanoparticles [21,39]. To this end, nanoflowers, nanostars, and other structures with higher curvature have been developed and tested as SERS substrates [39,40,41] (Figure 4). 

Apart from using the Au and Ag nanoparticles, after appropriate modification, in liquid phase assays, deposition of Au and Ag nanoparticles onto planar substrates or in situ synthesis has been exploited in order to perform solid phase assays. The deposition of metal nanoparticles can be performed by drop casting, spin-coating, or adsorption from a solution, while the in situ synthesis can be performed by a variety of methods such as colloidal lithography, e-beam lithography, electrochemical methods, etching and metal deposition, etc. [39,40,41,42,43]. Copper nanoparticles have also been implemented as SERS substrates since they have lower costs compared to Au and Ag nanoparticles but similar optical properties. Their use, however, is limited due to the fact that they are prone to oxidization under atmospheric conditions [44]. In addition to metals nanoparticles, the SERS effect has been reported for a high variety of other materials such as NiO, Cu_2_O, Ag_2_O, AgX (X = Cl, Br, I), ZnO, TiO_2_, α-Fe_2_O_3_, Si, Ge, graphene, and InAs/GaAs quantum dots [29,40]. The use of these materials for the fabrication of SERS substrates is gaining ground due to the lower cost of the reagents involved compared to Au and Ag and the compatibility of most of them with complementary metal-oxide semiconductor (CMOS) procedures.

### 2.2. SERS Labels

**Figure 5:**
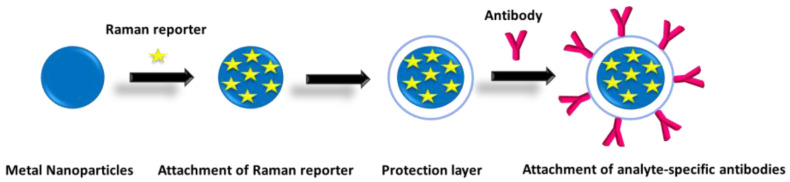
Procedure for the fabrication of a typical SERS label: at first, the metal nanoparticles (Au, Ag, etc.) were modified with the Raman reporter molecules, which are either molecules with thiol groups that readily react with the metal or are covalently bound onto the metal particles after chemical functionalization, a protection layer is then applied usually a silicon dioxide layer which after chemical functionalization provides for the attachment of antibody molecules either through adsorption or covalent bonding.

One of the most remarkable characteristics of detection methods based on SERS is that an analyte can be identified by its unique Raman spectrum, thus providing for its label-free detection. Unfortunately, this direct label-free detection is not possible when the analyte is present in the samples to be analyzed at very low concentrations or if it has a very weak Raman scattering signal. One method for inducing a strong SERS signal in the absence of an intrinsic signal from the analyte is to conjugate a Raman reporter molecule onto a plasmonic metal nanoparticle so as to create a SERS label [45]. As shown schematically in Figure 5, a typical SERS label consists of the metal nanoparticle, Raman reporter molecules, a protection layer, and the mean to bind the targeted molecules-analytes. In a nutshell, SERS labels are essentially Au or Ag nanoparticles or nanostructured surfaces that are co-functionalized with a Raman tag molecule and an analyte-specific biorecognition molecule (e.g., antibody, aptamer, lectin, antigen, etc.) to achieve both enhanced SERS signals and specific binding to targeted molecules-analytes. In such SERS labels, the SERS signal is enhanced by the localized SPR effect of the nanoparticles to which the Raman reporter molecules have been immobilized. The addition of one or more protecting layers around the metal nanoparticles aims to protect the Raman reporter molecules from the outside environment and/or provide anchor spots for the analyte biorecognition molecules [43].

In order for a “good Raman scatter” to be considered a “good SERS label,” the molecule must attach efficiently to the SERS substrate. Some molecules, especially those having a thiol group, have a strong chemical affinity for metal surfaces and are, therefore, easier to work with. Other mechanisms of probe attachment include electrostatic interactions, where charged probes adsorb on an oppositely charged substrate or the substrate is appropriately functionalized through the introduction of reactive groups that could be then used to covalently bind the Raman reporter molecules [46]. Other desirable properties of a SERS label are low water solubility and excitation in the near-infrared wavelength region. More specifically, the high water solubility of a SERS label may disrupt the bond between the label and the substrate, especially in cases where the attachment is based on electrostatic interactions, thus affecting the stability and reproducibility of the SERS measurements in aqueous samples. To this end, malachite green isothiocyanate (MGITC), 5,5′-dithiobis(2-nitrobenzoic) (DTNB), 4-nitrothiophenol (4-NTP), 4-mercaptobenzoic acid (MBA), rhodamine 6G and crystal violet, are considered as strong and stable SERS labels due to their high affinity with the metal substrates. Moreover, molecules containing alkyne moieties are considered good SERS labels due to their characteristic Raman peak at 2100 cm^−1^, where no other intrinsic peaks exist, providing high detection specificity [47]. It is also preferable for a label to be active in near-infrared, such as 3,3′-diethylthiatricarbocyanine (DTTC), due to the fact that near-infrared lasers provide negligible autofluorescence/absorption interference [48]. It should be noted that when the modified nanoparticles and nanostructured substrates are exposed to complex matrices such as the biological samples, their performance with respect to SERS detection is compromised due to a variety of physical and/or chemical phenomena that take place (e.g., corrosion, aggregation, unspecific absorption of biomolecules, etc.). In this case, the interaction between the nanomaterials and samples must be fully understood in order for the SERS-based detection to become a practical and reliable bioanalysis technique [5,49].

## 3. SERS-Based Immunosensors

SERS-based immunosensors combine immunoassays with surface-enhanced Raman scattering as the readout signal. An immunoassay is defined as an analytical method that takes advantage of the highly specific binding between an antibody and the antigen against which it has been developed for the quantitative determination of this antigen in a sample [46], where the antigen can be a low molecular weight synthetic or natural molecule or a high molecular weight protein molecule. To perform a SERS-based immunoassay, either the antibody or the antigen (or an antigen derivative) must be immobilized onto the SERS substrate, then the immunoreaction, i.e., the reaction between the antibody and the antigen in the sample, takes place. In general, the immunoassays performed in a SERS substrate follow the same formats and principles as standard microtiter plate immunoassays.

Thus, in accordance with the universal classification for immunoassays, SERS-based immunoassays could be classified into homogeneous or heterogeneous assays, competitive or non-competitive assays, and assays employing labels or label-free ones. In homogeneous immunoassays, there is no need to physically separate the bound from the free forms of the immunoreagents since the antibody–antigen binding modifies the signal enabling their discrimination [50]. Heterogeneous immunoassays, on the other hand, rely on the separation of the bound and free forms of the immunoreagents after the immunoreaction since the binding does not affect the signal. After separation, the analytical signal from the bound or free form of the immunoreagents is quantified and correlated to the analyte concentration in the sample [50]. 

**Figure 6:**
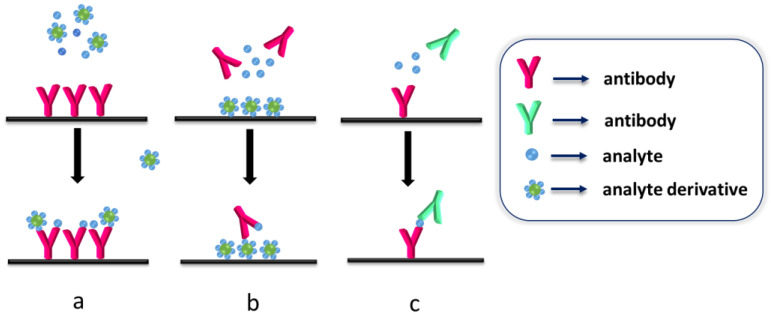
Schematic presentation of different immunoassay formats: (**a**) competitive immunoassay based on immobilized analyte-specific antibody for the binding sites of which compete for the analyte and an analyte-derivative (label), (**b**) competitive immunoassay based on immobilized analyte derivative that competes with the analyte for binding to the specific antibody binding sites, and (**c**) non-competitive immunoassay employing two antibodies, one immobilized that captures the analyte and the other in liquid-phase for bound analyte detection.

The application of a competitive or a non-competitive immunoassay format depends on the nature and the molecular weight of the antigen since it defines the number of available epitopes, i.e., the specific part of a molecule that the antibody binds to. Thus, for low molecular weight antigens that have only one epitope, a competitive immunoassay is the format of choice. As shown in Figure 6, there are two main competitive immunoassay configurations, one based on the competition of the analyte in the sample with the labeled analyte (or a labeled analyte derivative) for binding to an immobilized antibody (Figure 6a); and the second based on the competition between the immobilized analyte (or an analyte derivative) and the analyte in the sample for binding to a liquid phase antibody (Figure 6b) [51,52]. Due to the competitive immunoassay principle, the signal determined by the solid-phase bound reagents after completion of the immunoreaction is inversely proportional to the concentration of the analyte in the sample. The non-competitive immunoassay is suitable for antigens that have at least two epitopes, and therefore there are at least two antibodies that recognize these epitopes specifically [51,52,53]. One of the antibodies is immobilized onto the SERS substrate, and the immunoreaction between the two antibodies and the analyte results in “sandwich-like” immunocomplexes (Figure 6c) [51,52,53]. In this case, the analytical signal is proportional to the analyte concentration in the sample.

Finally, the SERS-based immunoassays could detect the targeted analyte either in a label-free format or by employing labels. Label-free immunosensing can rely on the analyte detection after directly binding to the antibody immobilized onto the SERS substrate or employing a non-competitive immunoassay with no labeled antibodies. On the other hand, the antibody or the antigen could be immobilized on a nanoparticle modified with a Raman reporter molecule and implemented in a competitive or non-competitive immunoassay format for the detection of the analyte. Label-based SERS immunoassays enable highly sensitive detection of the analyte via monitoring the spectra of the Raman reporter molecules as compared to respective label-free approaches [54,55].

## 4. Cancer Biomarkers Immunochemical Detection via SERS

Biomarkers are compounds that are used as indicators to differentiate normal from disease conditions, and their detection can increase the treatment success rates for many diseases. Biomarkers include low molecular weight molecules, such as steroid and thyroid hormones, lipids, etc., proteins including enzymes, structural proteins, protein-based hormones, etc., DNA fragments, and RNA molecules, as well as every molecule that its concentration in the biological fluids decreases or increases in response to a pathological condition. 

Especially in the field of cancer, biomarkers play a critical role in disease diagnosis, prognosis, and therapy monitoring; thus, there is a strong need for the development of highly sensitive and accurate diagnostic platforms for the detection of these biomarkers in biological fluids [56,57]. One of the approaches exploited to achieve this goal is the implementation of SERS-based immunochemical detection of protein cancer markers. In the following sections, the so-far reported SERS immunoassays for protein cancer marker detection are presented per targeted marker.

### 4.1. Prostate Specific Antigen (PSA)

Prostate cancer is the second leading cause of death from cancer in the male population worldwide. The most commonly used cancer biomarker for prostate cancer diagnosis and monitoring is prostate-specific antigen (PSA) [58]. PSA is a serine protease that belongs to the tissue kallikrein family, and it is produced by the prostatic epithelial cells in order to hydrolyze high molecular weight proteins produced by the seminal vesicles and allow the liberation of spermatozoa from the semen coagulum during ejaculation. Despite the fact that PSA is found in other tissues, including the breast, ovary, lung, pancreas, colon, kidney, and liver, in both men and women, its levels in human serum are low (<4.0 ng/mL in men; <0.004 ng/mL in women) unless a malignancy or pathological conditions is present. Thus, PSA levels in male serum above 10 ng/mL are indicative of a serious possibility of prostate cancer, whereas values between 4.0 and 10 ng/mL are not conclusive since they can be due to other pathological situations related to prostate such as prostatitis, benign prostate hyperplasia, etc. [58]. However, PSA is the most appropriate marker to monitor the relapse of prostate cancer after prostatectomy, and therefore it remains, despite its limitations, the most specific marker for prostate cancer. The determination of PSA levels in human serum samples is almost exclusively performed by immunochemical methods and especially by non-competitive immunoassays run on different platforms and instruments. Due to the significance of PSA for prostate cancer diagnosis and prognosis, there is a great interest in developing immunosensors for rapid and accurate PSA determination in human fluids [59].

To this end, one of the first reports regarding cancer markers immunochemical detection with SERS was about the determination of PSA through a non-competitive immunoassay onto a substrate patterned by microcontact printing to define areas modified with (3-aminopropyl)trimethoxysilane onto which Au nanoparticles could be attached [60]. The immobilized Au nanoparticles were then modified with an anti-PSA antibody, whereas Au nanoparticles modified with the Raman reporter molecule rhodamine 6G and an anti-PSA antibody were used as labels. Detection of PSA to concentration as low as 1 pg/mL was claimed, but a full evaluation of sensor performance was not conducted. 

SERS detection was combined with an enzyme immunoassay to detect PSA in human serum with a limit of detection (LOD) of 10^−9^ ng/mL [61]. This ultrasensitive detection was achieved by performing a non-competitive immunoassay in a 96-well microtiter plate using a detection antibody immobilized along with catalase onto Au nanoparticles. In the presence of PSA, the bound-to-wells catalase would oxidize its substrate, H_2_O_2_, and thus, upon the addition of aggregated Au nanoparticles modified with the Raman tag 4-mercaptobenzoic acid (4-MBA), a strong Raman signal was measured. On the other hand, in the absence or with low concentrations of PSA, most of the hydrogen peroxide was not consumed by the enzyme, and thus, dissolution of aggregated gold nanoparticles took place, and the Raman signal was reduced.

In another approach, silica-coated Ag nanorods modified with the Raman tag 4-MBA were optimized with respect to their morphology and Raman signal enhancement and then employed as labels in a non-competitive immunoassay for PSA with the capture antibody immobilized onto a quartz slide [62]. A LOD of 0.3 fg/mL was determined, and the linear working range extended up to 30 μg/mL.

A SERS-based microdroplet sensor for wash-free magnetic immunoassay of PSA has also been reported [63]. The sensor relied on embedding a magnetic bar into a microfluidic system for the separation of immunocomplexes formed by reacting magnetic beads modified with an anti-PSA antibody along with the sample and a second antibody immobilized onto Au nanoparticles labeled with the Raman tag malachite green isothiocyanate, from the free particles through droplet splitting. When PSA was present in the sample, more SERS labels were in the form of immunocomplexes and less in the free form. Thus, by determining the Raman signal from the free labels, the PSA concentration in the sample was indirectly determined. The LOD was 0.1 ng/mL, and the dynamic range was up to 200 ng/mL.

Another microfluidic device that was designed not to require external pumps was also implemented for the development of a SERS-based immunoassay for PSA using magnetic beads modified with the capture antibody and Au nanoparticles modified with the detection antibody and the Raman tag malachite green isothiocyanate [64]. The immunoreagents were mixed and reacted in a microfluidic device, and then, the immunocomplexes were separated by the free molecules by applying a magnetic field to the reaction chamber 5 min after their introduction. The LOD was determined to be 0.01 ng/mL, and the assay’s dynamic range extended up to 100 ng/mL, covering both the normal and prostate cancer range of concentrations, although no results from the analysis of human serum samples were demonstrated [64].

In another report, it was demonstrated that the addition of Ag nanoparticles on the surface of graphene oxide enhanced the Raman signal obtained from both the high-frequency disordered band (D-band) and tangential mode (G-band) bands of GO [65]. Such a surface was used as a substrate for the development of a non-competitive immunoassay for PSA in which the detection antibody was modified with biotin to enable the reaction with streptavidin labeled with glucose oxidase. After completion of the immunoreaction, the surfaces were incubated with the enzyme–substrate, i.e., glucose, and the hydrogen peroxide produced dissolved the deposited onto graphene silver nanoparticles. Thus, the Raman signal was reduced as the PSA concentration in the solution increased. The LOD achieved following this method was calculated at 0.23 pg/mL, while the assay dynamic range was from 0.5 to 5.00 pg/mL [65]. The SERS immunosensor developed was tested using a number of human serum samples (six in total), which were appropriately diluted prior to analysis so that their concentration fell within the assay’s dynamic range, indicating the potential of the method developed for the analysis of human serum samples.

A non-competitive SERS-based immunoassay for the quantitative detection of PSA was developed by combining Ag nanoparticle aggregates with an inbuilt Raman reporter molecule (2-naphthalenethiol) encapsulated in a polystyrene-block-poly(acrylic acid) layer and modified with an antibody against PSA with Au nanowires on a silica wafer also modified with an antibody against PSA [66]. By employing Ag nanoparticle aggregates instead of single Ag nanoparticles, an enhancement factor of 10 was achieved, which was further increased by a factor of 1.34 when a substrate with gold nanowires was employed instead of a plain silica surface. Thus, a LOD for PSA of 1 fg/mL was achieved combined with a very wide working range that extended up to 1 μg/mL (nine orders of magnitude) [66].

In a more complex approach, a non-competitive PSA immunoassay was developed using one antibody coupled to magnetic Au nanoparticles and another coupled to ZnO-Au nanocomplexes [67]. After immunoreaction, magnetic separation was applied to wash and concentrate the immunocomplexes. Then, the ZnO–Au nanocomplexes were dissolved with HCl, and the solution of Zn^2+^ ions created was applied to a silica wafer coated with Au nanoparticles modified with the Raman tag toluidine blue and then covered with a polyacrylamide gel containing a zinc finger peptide. Upon incubation with the solution of Zn^2+^ ions, the zinc finger peptide changed conformation, disrupting the polyacrylamide gel surface and allowing the detection of the Raman signal from the embedded tag in a concentration-dependent way. The proposed sensor could detect PSA in the range from 1 pg/mL to 10 ng/mL with a LOD of 0.65 pg/mL [67].

Microparticles with a core of Fe_3_O_4_ coated with a layer of TiO_2_ and then with Au nanoparticles (Fe_3_O_4_@TiO_2_@Ag microparticles) were investigated as a recyclable substrate for a SERS-based PSA immunoassay [68,69]. It was found that the photocatalytic degradation of the microparticles could be adjusted by tuning the thickness of the middle TiO_2_ shell and the density of the Ag nanoparticles at the outer surface, while the magnetic properties of the Fe_3_O_4_ core enhanced the photocatalytic activity of the microspheres [68]. PSA was covalently coupled onto these microspheres and was detected after binding to an anti-PSA antibody immobilized onto a slide. A LOD of 16.25 pg/mL was claimed, and a linear dynamic range from 0.1 ng/mL to 100 μg/mL [68]. Microspheres with the same structure have also been employed as labels in a non-competitive PSA immunoassay after their modification with the Raman tag 4-MBA and an anti-PSA antibody [69]. SiC sandpapers with sputtered Ag films were used as substrates after modification with an anti-PSA antibody. Through optimization of both the substrate and the labels, a LOD of 1.871 pg/mL was achieved [69].

**Figure 7:**
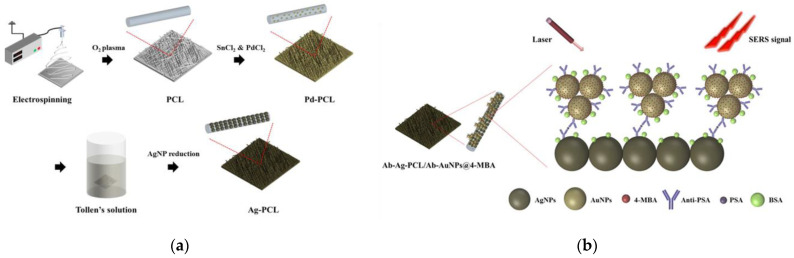
(**a**) Schematic configuration of Ag-PCL surfaces fabrication. (**b**) Illustration of an immunoassay for the detection of PSA with Ab-AuNPs@4-MBA SERS tag on the Ag-PCL surfaces prepared as shown in (**a**) [70]. Copyright 2019. Reproduced with permission from Elsevier B.V.

In another report, electrospun polycaprolactone fibers (PCL) decorated with Ag nanoparticles were implemented as substrates for a SERS-based immunoassay for the determination of PSA in combination with Au nanoparticles modified with 4-MBA (Figure 7) [70]. The large surface area of these substrates allowed the immobilization of larger numbers of antibody molecules compared to flat substrates, while their porous nature facilitated the reaction of immobilized antibodies with the antigen. Thus, a LOD of 1 pg/mL was achieved for a reaction time of 1 h [70].

In addition to immunoassays for SERS-based PSA determination, an aptasensor was proposed [71], which combined magnetic nanoparticles modified with an aptamer specific for PSA and Au nanoparticles modified with a complementary sequence and the Raman tag 4,4′-dipyridyl. After the reaction, the magnetic aptamer-mediated assemblies of Au nanoparticles were removed from the solution employing a magnet, and the Raman signal from the Au nanoparticles in the solution was detected. Thus, upon increasing the concentration of PSA in the sample, the SERS signal obtained from the supernatant was proportionally increased since, in the presence of PSA, the assembly of aptamer-modified magnetic nanoparticles with the Au nanoparticles was disrupted. The LOD achieved with this aptasensor was 5.0 pg/mL, with a linear range of up to 500 pg/mL. Furthermore, the sensor was applied for PSA determination in human serum samples demonstrating good specificity and satisfactory accuracy [71].

Another alternative binding moiety employed for PSA determination by SERS was based on molecularly-imprinted polymers (prepared using tannic acid as the functional monomer and diethylenetriamine as the cross-linker) deposited onto magnetic nanoparticles along with Au nanoparticles modified with an anti-PSA antibody and the Raman reporter 5,5′-dithiobis-(2-nitrobenzoic acid) [72]. The sensor LOD was 0.9 pg/mL, with a dynamic range from 3.2 pg/mL to 1 μg/mL.

PSA is encountered in human serum in two forms, the protein-bound, and the free form. Usually, the immunoassays developed for PSA determination target both the bound and free forms or the total PSA as it is termed since most clinical studies correlate the total PSA value with the diagnosis/monitoring of prostate cancer. Nonetheless, free PSA has also been used as a prostate cancer biomarker, and, in this frame, a SERS-based immunosensor for free PSA has been reported [73]. The immunosensor employed Au nanoparticles with an Au core modified with 1,4-benzenedithiol and covered with an Au layer. Embedding of the Raman tag 1,4-benzenedithiol prevented its interaction with other proteins in the sample, thus improving the stability and sensitivity of the probe. A LOD of 2.0 pg/mL was obtained, and the linear dynamic range was from 10 pg/mL to 10 ng/mL.

In Table S1, the analytical characteristics of all detection approaches for PSA determination by SERS-based sensors are summarized. Most of the reported SERS-based immunosensor for the detection of PSA have LODs of a few pg/mL that are well below the upper limit of the PSA normal range of 4 ng/mL. Nonetheless, this high detection sensitivity is very important since in prostectomized men, the values of PSA are close to zero, and any significant increase in its serum concentration might signify prostate cancer recurrence. It should also be noted that amongst the reports for SERS-based PSA detection, there is one with an outstanding LOD of 10^−9^ ng/mL or 1 ag/mL [61]. This is achieved by using as a label an enzyme that causes the in situ aggregation of Au nanoparticles modified with the Raman tag 4-MBA. Thus, a considerable signal enhancement is achieved compared with the rest of the reports, where the labels are not enzymatic. 

### 4.2. Alpha-Fetoprotein (AFP)

Alpha-Fetoprotein (AFP) is a plasma protein mainly found in human fetuses. Therefore, AFP levels in maternal serum during pregnancy have been correlated with severe complications in the growth of the fetus. In addition, AFP is produced at very low levels in healthy individuals, mainly from the liver (<10 ng/mL), and thus, the elevation of its concentration is indicative of malignant diseases and especially of primary hepatocellular carcinoma [74]. Thus, AFP is widely used for the diagnosis and monitoring of liver cancers.

**Figure 8:**
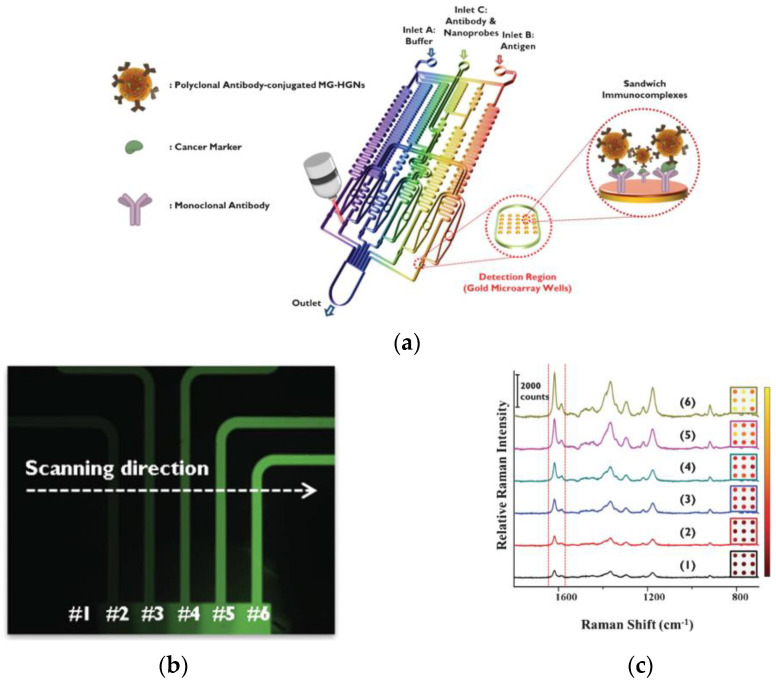
(**a**) Schematic of the microfluidic platform with embedded Au arrays for the SERS detection of AFP. (**b**) Fluorescence image indicating the automatic serial dilution of a dye solution in the six outlet channels of the microfluidic device shown in (**a**). (**c**) SERS spectra of a serially diluted AFP calibrator (with concentration decreasing from 6 to 1) obtained from the Au arrays at the six outlet channels of the microfluidic device shown in (**a**) [75]. Copyright 2012. Reproduced with permission from The Royal Society of Chemistry.

The first report regarding the implementation of SERS-based immunochemical determination of AFP used hollow Au nanospheres modified with an anti-AFP antibody as labels and an Au array incorporated into a microfluidic device as substrate (Figure 8a) [75]. The microfluidic design enabled the automatic dilution of calibrators or samples through microfluidic gradient generators and N cascade-mixing stages (Figure 8b,c). Thus, the total analysis time, from sample introduction to SERS detection, took about 60 min with a LOD of 1 ng/mL.

In another report, Au nanoparticles modified with 4-MBA were used as labels in a non-competitive immunoassay for AFP performed on top of a glass slide modified with Au nanoparticles [76]. The LOD was 100 pg/mL, and the linear dynamic range was 1 to 100 ng/mL. Nanorods made of NiCo_2_O_4_ and decorated with Ag nanoparticles were used as substrates for the immunochemical determination of AFP using as labels SiO_2_ microspheres coated with Ag nanoparticles and 4-MBA [77]. The particular substrate offered a strong SERS signal due to the immobilization of a large amount of homogeneous Ag aggregates on the one-dimensional nanorods. Thus, a LOD of 2.1 fg/mL was obtained. 

Enhancement of the Raman signal was also attempted through careful design of the Raman tags. Thus, Au nanospheres were coated with an Ag layer, an ultrathin silica shell, and finally with Au nanosphere satellites and modified with an antibody against AFP to be used as labels in a non-competitive assay in which the capture antibody was immobilized onto a nitrocellulose membrane [78]. Using the optimum nanospheres, a LOD of 0.3 fg/mL was achieved, and the method linear dynamic range was 1 fg/mL to 1 ng/mL. Similarly, a nitrocellulose membrane modified with an anti-AFP antibody was used as substrate in combination with another antibody immobilized onto Au/Ag nanostars [79]. The Ag deposition onto Au nanostars took place from the center outward, resulting in particles with a polyhedral shape. The Au/Ag nanostars were then modified with 4-MBA and covered with a SiO_2_ layer prior to antibody immobilization. It was shown that the Au/Ag nanostars provided a much higher SERS signal than the respective Au nanostars, and their resonance wavelengths could be tuned across a wide spectrum (from visible to near-infrared) by adjusting the Ag shell thickness. The implementation of these particles as labels led to a LOD of 0.72 pg/mL and a wide linear dynamic range (3 pg/mL–3 μg/mL).

In another report, a molybdenum disulfide (MoS_2_) sheet was modified with the anti-AFP antibody, while the detection antibody was labeled with rhodamine 6G [80]. After the completion of the immunoreaction, Au nanospheres or Ag-coated Au nanocubes were drop-cast onto the surface to enhance the Raman signal. It was found that the Ag/Au nanocubes provided higher signal enhancement compared to Au nanospheres. Thus, a LOD of 0.03 pg/mL was achieved with a linear dynamic range from 1 pg/mL to 10 ng/mL.

Apart from antibodies, molecularly imprinted polymers have been employed as binding moieties for the detection of AFP in biological fluids by SERS. In particular, a boronate-affinity molecularly imprinted polymer that binds glycoproteins has been used to develop a non-competitive assay for AFP [81]. Arrays of the molecular imprinting polymer were created on a glass slide to bind the AFP, while Ag nanoparticles modified with boronic acid were used as labels. In this way, AFP at concentrations ranging from 1 ng/mL to 10 μg/mL could be detected. The application of the sensor to determine AFP in human serum samples was demonstrated, while the use of a portable Raman spectrograph made the method suitable for on-site applications. The same approach was implemented to detect, in addition to AFP, the Lens culinaris agglutinin (LCA)-reactive fraction of AFP (AFP-L3), another marker of hepatocellular carcinoma, in a single run [82]. For this purpose, two molecular imprinted polymers were prepared, one recognizing the peptide sequence of AFP and a fucose-imprinted polymer that specifically recognized the AFP-L3 fraction. The two polymers were spotted onto a glass slide coated with Au nanoparticles as well as onto Ag nanoparticles that were also labeled with two different Raman tags to serve as labels. Following this approach, detection of AFP from 0.1 ng/mL to 10 μg/mL, and of AFP-L3 from 0.1 to 8 ng/mL was reported.

AFP-L3 was the targeted molecule in a report where a multi-layer comprised of Ag/Fe/Ag films was created on top of a monolayer of polystyrene colloidal particles and used as a SERS substrate after modification with an anti-AFP-L3 antibody and the Raman reporter 5,5′-dithiobis(succinimidyl-2-nitrobenzoic acid) to detect AFP-L3 at concentrations from 0.5 to 8 ng/mL [83]. 

Finally, in another approach for the simultaneous determination of AFP and AFP-L3 via SERS, Au nanoparticles modified with 5,5-dithiobis(succinimidyl-2-nitrobenzoate) and an antibody against AFP-L3 in combination with a silicon substate modified with Ag nanoparticles, 4-MBA and an antibody against AFP were used [84]. Thus, it was possible to discriminate the signal arising from the binding of total AFP to immobilized antibody but also detect the AFP-L3 fraction. Table S2 summarizes data on available SERS-based sensors for AFP detection. From these data, one can conclude that the SERS-based sensors developed for the detection of AFP have LODs and dynamic ranges that extend from sub fg/mL to ng/mL and are, therefore, suitable for the detection of AFP in human serum samples. The most sensitive detection is achieved using as labels Au nanoparticles that have been coated by an Ag layer on which the Raman tag 4-MBA was attached, and after covering the modified nanoparticles with a silicon dioxide layer, Au nanoparticles of smaller dimension than the initial ones were attached [78]. These multilayer nanoparticles that combine two noble metals considerably enhance the SERS signals compared to single metal layer nanoparticles. In addition, a nitrocellulose membrane was used as a substrate for capture antibody immobilization that also favors the attachment of higher amounts of antibodies with respect to planar non-porous substrates, e.g., glass slides [75,76].

### 4.3. Carcinoembryonic Antigen (CEA)

Carcinoembryonic antigen (CEA) refers to a family of glycoproteins that are expressed in various tissues and are implicated in a wide range of pathophysiological functions, including cell–cell adhesion, pregnancy, immunity, neovascularization, regulation of insulin homeostasis, and carcinogenesis [85]. Thus, it has been extensively used as a cancer marker primarily for colorectal cancer but also for other types of cancer, such as lung cancer [84]. 

Regarding CEA determination by SERS, the first report employed hollow Au nanospheres conjugated with the detection antibody and modified with a Raman tag (4-MBA) and silica-coated magnetic microspheres modified with capture antibody for the development of a two-step non-competitive immunoassay for CEA determination [86]. Magnetic microspheres of a diameter around 1 μm were synthesized, coated with silica, and modified with an amino-silane to allow covalent bonding of the capture antibody, whereas the hollow Au nanospheres were first modified with 4-MBA and mercapto-ethanol as blocking agent and then conjugated with the detection antibody. The sample was first mixed with the antibody-decorated magnetic microspheres, and after magnetic concentration and washing, a reaction with the immobilized onto hollow Au nanoparticles detection antibody was performed. In the presence of CEA, the Raman signal from the separated magnetic beads was increased in a concentration-dependent way as the Raman tagged-hollow gold nanoparticles were immunochemically attached to the magnetic microspheres. A LOD of 10 pg/mL was determined with the working range to extent up to 100 ng/mL. In addition, it was shown that by implementing hollow Au nanoparticles, the LOD was decreased by two orders of magnitude compared to that achieved with solid Au nanoparticles.

A non-competitive immunoassay for CEA was also developed using antibody-coated nanoprobes consisting of a magnetic nickel−iron core and an Au shell in combination with Au nanoparticles modified with 4-MBA and a second antibody [87]. The immunoreaction and detection were performed in a microfluidic channel, and the LOD was determined to be about 0.1 ng/mL. In another report, Au nanoparticles were modified with 4-MBA and an antibody against CEA and were used in combination with Fe_2_O_3_@Au, also modified with an antibody against CEA, to develop a non-competitive SERS-based immunosensor [88]. The method was successfully applied to the determination of CEA in human serum, and the results showed good selectivity and sensitivity with a LOD of 0.1 ng/mL. In addition, the values determined in human serum samples were in good agreement with those obtained for the same samples with conventional CEA determination methods [88]. Another non-competitive immunoassay for CEA based on SERS detection was also realized into a microfluidic chip that comprised four inlets, two chaotic mixers, a detection chamber, and an outlet [89]. The assay was performed in two steps; first, the raw blood sample was introduced into the chip via Inlet 1, and the antibody-coated magnetic nanoparticles were introduced via Inlet 2. After mixing and reaction, the immunocomplexes were mixed with the antibody-coated Au nanoparticles labeled with 4,4′-bipyridine introduced via Inlet 3 and then flowed to the detection chamber, where they were washed to remove the blood cells prior to the measurement. The sensor could detect CEA in whole blood at concentrations ranging from 1 pg/mL to 1 μg/mL. Au nanoparticles modified with polydopamine and decorated with the Raman tag Nile blue were used after the attachment of an anti-CEA antibody as labels in a non-competitive assay for CEA for both SERS and electrochemical detection [90]. It was shown that the performance of the electrochemical immunoassay was better than that of the SERS immunoassay. For the SERS assay, the LOD was 1.38 ng/mL, and the linear dynamic range was 2 to 100 ng/mL. A SERS-based immunosensor that included as labels MoS_2_ nanoflowers decorated with Au nanoparticles modified with 4-MBA and Fe_3_O_4_/Au nanoparticles incorporated onto delaminated Ti_3_C_2_T_x_ MXene sheets, i.e., two-dimensional nanocrystals produced by exfoliation of Ti_3_AlC_2_, as SERS substrate has also been reported. A LOD of 0.033 pg/mL was determined with a liner working range from 0.0001 to 100.0 ng/mL [91]. The sensor was also evaluated by analyzing blood plasma samples and performing recovery experiments in those samples.

As in the case of other cancer markers, molecularly imprinted polymers have also been employed as binders for the determination of CEA by SERS [92,93,94,95]. Thus, Au nanoparticles were modified with 4-mercaptobenzonitrile prior to the development of Ag aggregates and reaction with a mixture of 4-aminothiophenol and 4-mercaptophenylboronic acid to allow covalent coupling of anti-CEA antibodies [92]. The resulting nanoparticles were spread on a surface, reacted with CEA, and embedded onto the polymer, made by reaction of dopamine with m-aminophenylboronic acid monohydrate and ammonium persulfate, to create the solid-phase binder after removal of bound CEA, while Au nanoparticles modified with ethynylbenzene, covered with a poly-dopamine layer and conjugated to an anti-CEA antibody were implemented as labels. The signal from the embedded nanoparticles was used as an “internal standard” to normalize the Raman signal fluctuations. Thus, a LOD of 0.064 pg/mL with a linear detection range of 0.1 pg/mL–10 μg/mL was reported. In another report, a molecularly imprinted polymer, synthesized using a mixture of 15% aminopropyltriethoxysilane (APTES), 15% 3-ureidopropyl-triethoxysilane (UPTES), 30% isobutyltriethoxysilane (IBTES) and 40% tetraethyl orthosilicate (TEOS), was applied over a layer of self-assembled Au nanoparticles as well as to Ag nanoparticles that served as Raman tags [93]. A LOD of 100 pg/mL was reported, along with a linear dynamic range of 1 ng/mL–10 μg/mL.

Since CEA is a glycoprotein, the boronate-affinity molecularly imprinted polymer that was used for the determination of AFP was also applied [94]. The polymer was synthesized on a glass slide using 4-vinylbenzeneboronic acid as the functional monomer, ethylene glycol dimethacrylate as the crosslinking agent, and ethylene glycol and cyclohexanol as porogens, whereas Au nanoparticles labeled 4-mercaptophenylboronic acid were bound to CEA after its capture by the polymer. Following this method, CEA could be quantified in spiked serum with a LOD of 0.1 ng/mL and over a concentration of 1 mg/mL. Finally, a molecularly-imprinted polymer was combined with an antibody for SERS detection of CEA [95]. The polymer was synthesized by electropolymerization on top of Au-based screen-printed electrodes using gallic acid as a monomer in the presence of CEA. For the SERS detection, Au nanostars coupled to 4-aminothiophenol and an antibody against CEA were implemented. The LOD was determined to be 1.0 ng/mL, and the linear range reached up to 1000 ng/mL. Data presented in Table S3 regarding SERS-based sensors for CEA detection show that the most sensitive detection with a broad assay dynamic range is achieved with a liquid phase non-competitive immunoassay where MoS_2_ nanoflowers decorated with Au nanoparticles and modified with 4-MBA and an anti-CEA antibody were combined with Fe_3_O_4_/Au nanoparticles incorporated onto delaminated Ti_3_CT_x_ MXene sheets prior to attachment of a second anti-CEA antibody [91]. Another approach that provided high detection sensitivity used a molecularly imprinted polymer as a solid-phase binder in combination with antibody-modified Au nanoparticles [92], demonstrating the ability of binding moieties other than antibodies to be implemented in sensitive biomarker assays.

### 4.4. Carbohydrate Antigen 125 (CA125)

Carbohydrate or cancer antigen 125 (CA125) is one of the earliest identified biomarkers for ovarian cancer and remains the gold standard for both the diagnosis and monitoring of patient response to therapy as well as for the detection of relapse of ovarian cancer in hysterectomized patients [96,97], despite the fact that elevated CA125 blood serum levels can be found in a great variety of benign and pathological conditions [68]. CA125 is a very large (>1 MDa) mucin-like molecule with an N-terminal domain and up to 60 repeating subunits, each containing an identical sequence of 156 amino acids [96,97]. Due to its wide application as a biomarker for ovarian cancer diagnosis, CA125 detection based on SERS has also been explored.

Thus, there is a report for direct SERS detection of CA125 in human plasma samples by mixing Ag nanoparticles with the sample and depositing them on aluminum foil slides [98]. The results obtained by the SERS method were compared to those obtained for the same samples by Raman spectroscopy. The clinical sensitivity and specificity were substantially high in both SERS (87% and 89%, respectively) and Raman measurements (94% and 96%, respectively). Despite the excellent performance of the method regarding the discrimination of patients from non-patients, data about the analytical sensitivity and working range are not provided.

In another report, an antibody against CA125 was immobilized onto Au nanoparticles covalently coupled to a glass substrate modified with an amino-silane [99]. Upon binding of CA125 onto immobilized antibody, alterations in the Raman spectrum were observed, allowing its label-free determination at nM concentration levels, without, however, a complete analytical evaluation of the method.

In conclusion, the two reports for SERS-based CA125 immunosensors do not provide enough analytical data for a full evaluation and comparison despite the widespread application of this molecule as a cancer biomarker.

### 4.5. MUC4

MUC4 is a protein of the mucin family that is overexpressed in several pancreatic cancers, and patients with high levels of MUC4 are considered to have poor prognoses [100]. MUC4 is not detected in normal pancreas and chronic pancreatitis. Due to that, there are some reports regarding MUC4 determination with SERS-based methods.

In the first report, silicon wafers covered with an Au layer and modified with an antibody against MUC4 were combined with Au nanoparticles modified with 4-nitrobenzenethiol and a second anti-MUC4 antibody to develop a non-competitive immunoassay for MUC4 [101]. The LOD achieved was 33 ng/mL, and the dynamic range was up to 10 μg/mL. The assay was used to determine MUC4 in cell lysates and human serum samples. The same approach was applied to another cancer marker of the mucin family, Ca19-9, with a LOD of 0.8 U/mL and a dynamic range of up to 10 U/mL. 

Atomically smooth mica was used as substrate after Au deposition to immobilize an anti-MUC4 antibody, while Au nanoparticles decorated with 4-nitrobenzenethiol and another anti-MUC4 antibody were used as labels [102]. It was shown that the assay repeatability and sensitivity could be improved if, after the immunoassay, the immunocomplexes were covered by a thin film of polydimethylsiloxane as a protective layer against photo-irradiation. Although the assay was applied to detect MUC4 in human serum samples, analytical evaluation in terms of LOD and dynamic range was not performed.

To improve the spot-to-spot variation in the SERS signal, Raman mapping was applied onto a silicon substrate where Au nanoflowers were deposited and modified with an anti-MUC4 antibody [103]. The same Au nanoflowers were modified with 4-MBA and another antibody and used as labels in a two-step, non-competitive assay. The method provided a LOD of 0.1 ng/mL and a liner range of up to 10 μg/mL. Table S4 summarizes these data regarding MUC4 detection with SERS-based immunosensors; as shown, there is only one report referring to MUC4 detection in human serum, which, however, provides only preliminary results [102]. From the other two reports, a much lower LOD (330 times) is achieved when Au nanoflowers were used both for the preparation of the substrate and the label [103] compared to a flat gold substrate combined with gold nanoparticles [101]. This result verifies the general observation that SERS labels with some nanostructure enhance the Raman signal more efficiently than the respective smooth nanoparticles.

### 4.6. Human Epididymis Protein 4 (HE4)

Human epididymis protein 4 (HE4) is a relatively new marker that is used for the diagnosis of ovarian cancer but also to forecast the optimal cytoreduction after surgical treatment as well as to predict response to chemotherapy [104,105,106]. HE4 is a low molecular weight (approx. 31 KDa) glycoprotein originally found in the epithelial cells of the human epididymis. It is considered a more specific clinical marker, compared to CA125, for the diagnosis of ovarian cancer at an early stage with a serum cut-off level of 70 pmol/mL, above which there is a high suspicion of ovarian cancer [105]. 

There are only two reports regarding the determination of HE4 with SERS [107,108]. The first method employed Au nanoparticles modified with 4-MBA and an anti-HE4 antibody and magnetic core (Fe_3_O_4_) Au nanoparticles also modified with an anti-HE4 antibody [107]. The immunocomplexes were concentrated after the assay using a magnet to facilitate the SERS measurements. Following this method, a LOD of 100 fg/mL was demonstrated, along with a dynamic range from 1 pg/mL to 10 ng/mL. In addition, the Fe_3_O_4_/Au nanoparticles could be regenerated by treatment of the immunocomplexes with an acidified methanol solution and reused. In the second report, a single-crystalline Au nanoplate was used as a substrate for an anti-HE4 antibody attachment through thiol-modified protein G in combination with Au nanoparticles modified with the Raman reporter malachite green isothiocyanate and an anti-HE4 antibody [108]. A LOD of 0.31 fg/mL (10^−17^ M) was reported with a dynamic range up to 31 ng/mL (10^−9^ M). These data regarding SERS-based immunosensors for the detection of HE4 are also summarized in Table S4. The second report [108] provided a more than 300 times enhancement of LOD with respect to the first one [107], despite the fact that in the second approach, the immunoreaction was performed in the solid phase, whereas in the first, it was in the liquid phase. Thus, this difference in analytical performance could be ascribed either to the higher binding affinity of the antibodies used in the second report or to the more efficient attachment of these antibodies to substrate and label, respectively.

### 4.7. Other Cancer Biomarkers

Since cancer is a poly-parametric disease with many different types, etiologies, and pathologies, several molecules have been investigated as possible markers aiming for early specific diagnosis. To this end, SERS-based methods of detection have been employed for the determination of other protein-based cancer markers onto biological fluids.

Growth factors, such as vascular endothelial growth factor (VEGF), are such markers. VEGF has been related to tumor-associated angiogenesis [109]. The SERS-based detection of this marker was accomplished by immobilizing a VEGF-specific antibody onto an Au triangle nanoarray to detect the analyte in a non-competitive immunoassay using Au nanostars modified with malachite green isothiocyanate and an antibody against VEGF. A LOD of 7 fg/mL was obtained with a dynamic range of 0.1 pg/mL–10 ng/mL. In addition to growth factors, the detection of their cell receptor in the blood is an indication of malignancy. Thus, human epidermal growth factor receptor 2 (HER2) is an important biomarker for breast cancer diagnosis and therapy since it is overexpressed in 20–30% of breast cancer patients [110]. The determination of HER2 in human blood serum was performed using Au/Ag nanoshells modified with malachite green isothiocyanate or fluorescent tags and coated with silica to protect the attached tags. These labels were modified with an antibody and used in a non-competitive assay with the capture antibody attached to Au electrodes incorporated into a microfluidic device. It was shown that by utilizing an alternative current electrohydrodynamic device, the capture kinetics were considerably improved, resulting in a 40 min assay with a LOD of 10 fg/mL [110]. 

As mentioned in the previous section, CA19-9 is another cancer marker belonging to the mucin family, and its levels in serum have been correlated with the diagnosis and/or prognosis of colorectal and pancreatic cancer. In addition to Ref. [101] discussed above, CA19-9 was immunochemically determined on F_3_O_4_ particles coated with a layer of TiO_2,_ onto which Au seeds were adsorbed to give rise to Au nanoparticles [111]. The Au nanoparticles were then modified with an antibody against CA19-9 and used to couple the CA19-9 from the sample, followed by a reaction with an anti-CA19-9 antibody modified with 4-MBA. The LOD was 5.65 × 10^−4^ IU/mL, and the linear dynamic range was 0.001 to 1000 IU/mL. However, the great advantage of the method was that by exposure to visible light, the F_3_O_4_/TiO_2_/Au could be regenerated and reused.

The transcription factor p53 has a central role in maintaining genome integrity, thus, preventing cancer development from random mutations. This onco-suppressing activity is lost when mutations on the gene encoding p53 take place, and thus, both the wild-type and mutant forms of p53 are useful cancer biomarkers [112]. To this end, SERS-based immunoassays for both the wild-type p53 and p53R175H, which is one of the most frequent tumor-associated mutants of p53, have been reported [112]. The method employed Au nanoparticles decorated with the specific antibodies through a diazonium compound linker as labels for detection of both p53 and p53R175H mutant through a direct binding assay realized onto silicon wafers modified with amine-silane and glutaraldehyde to covalently couple the analyte at concentrations as low as 10^−17^ M, and with a dynamic range from 10^−17^ to 10^−10^ M, both in buffer and serum.

Squamous cell carcinoma antigen (SCCA), a marker for diagnosis of cervical cancer, has also been detected with a SERS-based immunoassay [113]. For this purpose, polydopamine resin microspheres were coated with Au nanoparticles and then coupled with a monoclonal antibody against the antigen. These nanoparticles were used in combination with hollow Au nanocages modified with 4-MBA and a polyclonal antibody against the squamous cell carcinoma antigen to develop a non-competitive immunoassay. A LOD of 8.03 pg/mL in peripheral blood was reported with a dynamic range from 10^−5^ to 10^−10^ M. Another potential biomarker for cervical cancer detection is B7 homolog 6 (B7-H6) protein [114]. The SERS assay for the detection of B7-H6 explored an approach to suppress serum fouling through modification of Au-coated silicon substrates with a self-assembled monolayer of zwitterionic L-cysteine prior to their modification with the NKp30 receptor protein that captured the B7-H6 from blood serum. Then, Au nanoparticles functionalized with ATP as a Raman tag, and an antibody against B7-H6 were implemented to detect the surface-bound analyte. The LOD was determined to be 10^−4^ M or 10.8 fg/mL, which is at least 100 times lower than the concentration encountered in cancer patients.

Human carboxylesterase 1 is a member of the serine hydrolase superfamily, and it has been correlated with the occurrence of hepatocellular carcinoma [114]. The SERS immunosensor developed for the determination of human carboxylesterase 1 is based on raspberry-like magnetic nanocomposites consisting of Fe_3_O_4_/SiO_2_/Ag and functionalized with an antibody against the analyte as a substrate and Ag nanoparticles modified with 4-MBA and an analyte-specific antibody as label [115]. The assay dynamic range was from 0.1 ng/mL to 1.0 mg/mL, and the LOD was 0.1 ng/mL. The sensor was applied for the detection of the analyte in human serum without complicated sample pre-treatment.

Another potent cancer biomarker for which a SERS-based immunoassay has been developed is galectin-3-binding protein (LGALS3BP), also known as 90K, a protein involved in tumor growth and progression [116]. Silicate glass slides coated with a thin Au layer, composed of stochastic nanostructure feature fragments from tens of nm to a few mm, were functionalized with an analyte-specific antibody and used to detect the analyte upon its binding with a LOD of 15 ng/mL. The assay was employed to detect the analyte in human serum samples after appropriate dilution [116].

Ferritin has also been used as a marker for the early diagnosis, therapy, and tracing of liver cancer. For its detection through SERS, mesoporous hybrid SiO_2_ particles were coated with Au nanoparticles and then modified with 4-MBA and a ferritin-specific antibody to be used as labels in a non-competitive assay performed on sandpaper modified with Ag nanoparticles and functionalized with an anti-ferritin antibody [117]. It was shown that the implementation of the hybrid particles offered higher enhancement of the Raman signal compared to solid SiO_2_ particles. Thus, following this approach, a LOD of 31.6 fg/mL with a dynamic range from 1 pg/mL to 10 μg/mL was achieved. 

In addition to molecular biomarkers, extracellular vesicles or exosomes that participate in the communication between cancerous cells have been proposed as biomarkers for cancer diagnosis and prognosis [118,119]. Thus, nanorods with an Au core and an Ag shell were modified with the Raman tag 5,5′-dithiobis(2-nitrobenzoic acid) and an antibody against exosomes and used as labels for the determination of serum exosomes in combination with magnetic Fe_3_O_4_ nanoparticles covered with a silica shell and functionalized with an antibody against the targeted exosomes [118]. The immunocomplexes were separated from the reaction mixture using a magnet, and the Raman signal was determined, providing a LOD of 1200 exosomes (268 aM). In another report, an approach to simultaneously detect multiple protein biomarkers expressed on cancer-derived small extracellular vesicles was developed [119]. In particular, antibodies against three surface receptors, glypican-1, epithelial cell adhesion molecules (EpCAMs), and CD44 variant isoform 6 (CD44V6), were coupled to Au nanoparticles modified with different Raman tags and then with specific antibodies against the three molecules and used to detect extracellular vesicles from different cancer cells through a non-competitive immunoassay in combination with magnetic beads functionalized with an antibody against another receptor, CD63 [119]. The method LOD was 2.3 × 10^6^ particles/mL, and its dynamic range was up to 2.3 × 10^8^ particles/mL. Extracellular particles from different cancer cells have been detected following this approach. 

Aptamers have also been employed for the non-competitive determination of cancer markers using a combination of two Raman tags. Thus, α-thrombin was determined using Au nanoparticles labeled with the 4-nitrobenzenethiol and modified with an aptamer in combination with Au film functionalized with another aptamer and labeled with methylene blue as a substrate [120]. Following this approach, it was possible to discriminate signals due to specific binding from the signals due to the non-specific binding of labels. A LOD of 86 pM for thrombin was determined, and a dynamic range of up to 1 nM. The same principle was used to detect tumor necrosis factor-α (TNF-α) with the difference that instead of using two aptamers, an antibody was attached to the Au surface and an aptamer was used for detection. The LOD, in this case, was 0.07 nM, and the dynamic range extended to 1.2 nM.

BRCA1 protein (encoded by Breast Cancer Associated gene 1) is a tumor-suppressor molecule that plays a critical role in the development of hereditary breast cancer, and therefore it is considered a very specific marker for this disease. The assays developed based on SERS substrates for BRCA1 detection aimed at specific peptides and were realized by employing a modular microfluidic chip that enabled filtering of the targeted peptides from human serum, concentration, and detection [121,122]. Serum filtering was achieved by passing the mixture through a membrane with a cut-off of 12–14 kD, and then the filtered samples were driven to the SERS surface consisting of Ag grains, where they were adsorbed, enabling their direct detection by Raman spectroscopy. A LOD of 0.1 ng/mL was determined, which is considered satisfactory for BRCA1 protein detection in early breast cancer diagnosis. Table S5 summarizes these data about the detection of different cancer markers by SERS biosensors.

### 4.8. Interleukins

The cancer markers discussed in the previous section were high molecular weight compounds that are, in most cases, secreted almost exclusively from the cancerous cells. Nonetheless, other compounds, such as cytokines, have been used as biomarkers for cancer diagnosis. Cytokines are non-specific markers for the diagnosis of malignancies since an increase in their serum levels takes place in several inflammatory diseases and pathological situations, including cancer. 

Thus, a SERS-based immunoassay for interleukin-6 (IL-6) was developed employing Au/Ag nanoshells that were hydrophilically stabilized by coating their surface with a self-assembled monolayer of 5,5′-dithiobis(2-nitrobenzoic acid) molecules comprising terminal mono- and tri-ethylene glycol [123]. These stabilized particles were used as labels in a non-competitive immunoassay for IL-6, with the second antibody immobilized onto a glass slide. A LOD of 1 pg/mL was obtained with a dynamic range of up to 1 μg/mL. 

Interleukin 8 (IL-8) is another cytokine that plays an important role in tumor growth and angiogenesis, and it is overexpressed in several human tumors, including gastric cancer and breast cancer [124]. The SERS-based immunoassay developed for IL-8 detection implemented highly-branched Au nanoparticles functionalized with an anti-IL-8 antibody as substrate and Au nanocages modified with 4-MBA and anti-IL-8 antibody as a label. The linear dynamic range was from 10 pg/mL to 1 μg/mL, and the LOD was 6.04 pg/mL. 

The simultaneous detection of three interleukins, IL-6, IL-8, and IL-18, by SERS has been achieved using an Ag-Au substate incorporated into a microfluidic device and functionalized with specific antibodies for the three molecules in combination with detection antibodies coupled to Au nanoparticles labeled with three different Raman tags, i.e., 5,5′-dithio-bis(2-nitro-benzoic acid), fuchsin, and 4-MBA [125]. The three analytes were detected either in parallel or simultaneously. Principal component analysis was applied for discrimination of the signal corresponding to each one of the three Raman tags and, consequently, of the three analytes during their simultaneous detection. The LODs achieved were 2.3 pg/mL, 6.5 pg/mL, and 4.2 pg/mL in the parallel detection approach, and 3.8 pg/mL, 7.5 pg/mL, and 5.2 pg/mL in the multiplexed detection method for IL-6, IL-8, and IL-18, respectively. From the data regarding the analytical performance of SERS-based immunosensors for interleukins detection (summarized in Table S6), it is apparent that the three reports achieved LODs in the concentration range of a few pg/mL. In particular, the two reports regarding the single-analyte detection of IL-6 [123] and IL-8 [124] also demonstrated a wide dynamic range of up to 1 μg/mL. On the other hand, the report about the simultaneous determination of three interleukins (IL-6, IL-8, and IL-18) [125] demonstrated compatible detection sensitivity for a somewhat less wide dynamic range, which is, however, appropriate for the detection of these markers in human serum.

### 4.9. Multiplexed Cancer Markers Detection by SERS

Cancer diagnosis based on the determination of markers in biological fluids, mainly blood, has the limitation that most of the molecules used as markers are not specific for cancer, since their levels in the blood could also change due to a variety of pathological conditions. The determination of more than one marker, preferably in a single run, could increase the diagnosis validity and even provide for differential diagnosis between cancer and other pathological conditions as well as between the different cancer types. Thus, analytical techniques that provide for multiplexed markers determination in a single run are in demand. SERS-based detection could be easily adopted for multiplexed biomarkers determination either by scanning a surface where different binding molecules have been immobilized onto spatially discrete locations of the substrate or by using labels with discrete Raman spectra.

One of the first reports for simultaneous determination of cancer markers by SERS focused on the immunochemical determination of AFP and angiotensin by immobilizing the capture antibodies onto a micropatterned Au film and using hollow Au nanospheres modified with the respective detection antibodies and the Raman tag malachite green isothiocyanate [126]. The LODs achieved were 0.1 pg/mL and 1.0 pg/mL for angiogenin and AFP, respectively, and the assays dynamic range was up to 0.1 μg/mL for both analytes. 

CEA and AFP were the targeted lung cancer biomarkers in another report in which their simultaneous determination was accomplished by modifying hollow Au nanospheres with antibodies against CEA or AFP and malachite green isothiocyanate and X-rhodamine-5-(and-6)-isothiocyanate, respectively [127]. Magnetic beads also modified with antibodies against CEA or AFP were implemented as supports for the detection of both markers in blood serum using a single laser at concentrations up to 100 ng/mL. 

A parafilm mask was applied onto an Au-coated slide to define spots for the immobilization of antibodies against carbohydrate antigen 19-9 (CA 19-9) and matrix metalloproteinase-7 (MMP-7), two pancreatic cancer markers [128]. Au nanoparticles modified with antibodies against the two markers and 5,5′-dithiobis(succinimidyl-2-nitrobenzoate) were employed as labels in non-competitive immunoassays of the two markers with LODs of 2.28 pg/mL for MMP-7 and 34.5 pg/mL for CA19-9 and dynamic ranges up to 6 ng/mL for MMP-7 and 18 ng/mL for CA19-9, respectively.

**Figure 9:**
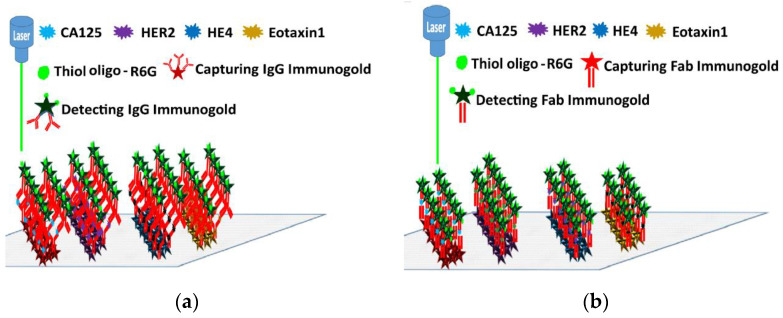
Illustration of sandwich assay format for the multiplex detection of CA125, HER2, HE4, and eotaxin 1, employing (**a**) whole IgG molecules or (**b**) Fab fragments both for capture and detection of the targeted analytes [128]. Copyright 2015. Reproduced with permission from Elsevier B.V.

A SERS-based microfluidic biosensor was applied for the simultaneous detection of breast cancer biomarkers, CA125, HER2, HE4, and eotaxin-1, in human serum samples [128]. The microfluidic reaction chamber surface was modified with Au nanostars on which the specific antibodies were attached, while Au nanostars modified with the Fab fragments of specific antibodies and Rhodamine 6G were used as labels (Figure 9). The LODs were 15 fM for CA125, 17 fM for HER2, 21 fM for HE4, and 6.5 fM for eotaxin-1, and the dynamic range for all markers was up to 10 pM [129]. 

Three breast cancer biomarkers, CA15-3, CA27-29, and CEA, have also been simultaneously detected in an antibody array on a quartz slide using as labels Au nanostars modified with 4-nitrothiophenol and embedded in SiO_2_ prior to the attachment of detection antibodies [130]. The LODs determined were 0.99 U/mL, 0.13 U/mL, and 0.05 ng/mL for CA15-3, CA27-29, and CEA, respectively, and linear dynamic ranges from 0.1 U/mL to 500 U/mL for CA15-3 and CA27-29, and 0.1 ng/mL to 500 ng/mL for CEA.

Photonic crystal beads of different sizes composed of periodically arranged monodisperse nanoparticles of SiO_2_ have been decorated with Ag particles and then modified with antibodies specific for CEA and AFP and used as labels in non-competitive assays for the two markers in combination with Ag nanoparticles also modified with antibodies against the two markers [131]. The simultaneous detection was based on the fact that the reflection peak positions differ for silica nanoparticles of different diameters. Thus, it was possible to detect in a single run CEA and AFP with LODs of 6.6 × 10^−6^ and 7.2 × 10^−5^ ng/mL, respectively. 

A multiplexed electrochemical and SERS-based immunoassay for CEA and cytokeratin-19 was realized using aminosalicylic acid-based resin microspheres modified with thionine and Nile blue A, that provided both strong SERS and electrochemical redox signals [132]. The microspheres were coated with Au nanoparticles, and the detection antibodies were attached. The capture antibodies were immobilized on electrodes modified with chitosan-stabilized Au nanoparticles. This approach led to LODs of 0.01 ng/mL and 0.04 ng/mL for CEA and CK-19, respectively, and a dynamic range for both analytes from 0.05 to 80 ng/mL.

For the simultaneous detection of CEA, AFP, and CA 125, three thiol compounds, 3-methoxybenzenethiol, 2-methoxybenzenethiol, and 2-naphthalenethiol were selected as Raman tag molecules to be incorporated in hybrid multilayered nanoshells prepared by the assembly of small Ag nanoparticles at the surface of silica particles using poly(ethyleneimine) [133]. After the attachment of respective antibodies, these particles were combined with superparamagnetic Fe_3_O_4_/SiO_2_ particles, also modified with antibodies specific against the three cancer markers. The LOD and dynamic range for CEA were mentioned and were 0.1 pg/mL, and 0.1 pg/mL to 1 ng/mL, respectively.

CEA and neuron-specific enolase (NSE), two lung cancer markers, have been simultaneously determined using SERS tags prepared by surface modifications of flower-like gold nanoparticles with Raman molecules and specific antibodies, while magnetic nanoparticles modified with mixed antibodies are used as capturing substrates for separation of immunocomplexes from the reaction mixture [134]. The LODs of CEA and NSE in human serum were 1.48 pg/mL and 2.04 pg/mL, respectively, and the dynamic range was from 1 fg/mL to 1 ng/mL for both markers.

A SERS substrate was prepared by sputtering an Ag film on SiC sandpaper which was then modified with specific antibodies against PSA, AFP, and CA19-9 for their determination in human serum using as labels Si nanoparticles coated with SiO_2_ and coupled to antibodies after modification with 3-(aminopropyl)trimethoxysilane [135]. The LODs achieved for PSA, AFP, and CA19-9 in human serum were 1.79 fg/mL, 0.46 fg/mL, and 1.3 × 10^−3^ U/mL, respectively. SiC sandpaper with a sputtered Ag film was also employed to simultaneously detect PSA, prostate-specific membrane antigen, and human kallikrein 2, aiming to discriminate between prostate cancer and benign prostate hyperplasia [136]. This substrate was combined with Ag nanoparticles labeled with 4-MBA and specific antibodies as Raman labels in non-competitive assays that achieved detection of PSA, prostate-specific membrane antigen, and human kallikrein 2 at concentrations as low as 0.46 fg/mL, 1.05 fg/mL and 0.67 fg/mL respectively, while the dynamic range was up to 1 ng/mL for all markers. A similar approach involved ordered Au nanohoneycomb arrays as substrate and Au nanostars modified with either 4-MBA or DTNB and the respective specific antibodies for the multiplex detection of CEA and AFP [137]. The LODs achieved were 0.44 and 0.40 ng/ml for CEA and AFP, respectively, with a linear dynamic range from 0.5 to 100 ng/mL.

To define the reaction area for the simultaneous detection of CA153, CA125, and CEA, microchannels were created on a chip made of polydimethylsiloxane (PDMS), through which the Ag nanoparticles and the specific antibodies were applied onto a glass slide [138]. Ag aggregates labeled with three different Raman tags and specific antibodies for the three analytes were then run using a three-channel microfluidic positioned at a right angle with respect to the first one. Thus, it was possible to determine for each analyte the specific and the non-specific signal. The LODs of CA153, CA125, and CEA in serum were 0.01 U/mL, 0.01 U/mL, and 1 pg/mL, respectively, and the dynamic ranges up to 1000 U/mL for CA153 and CA125, and 100 ng/mL for CEA.

PSA and AFP were simultaneously detected on an Au-film hemisphere array created by Au deposition on a layer of plasma-etched polystyrene nanospheres upon a silicon substrate [139]. Silica beads coated with Ag nanoparticles were modified with 4-MBA or 4-nitrothiophenol prior to the coupling of PSA and AFP antibodies to be used as labels. The assay developed had a linear dynamic range from 10 fg/mL to 400 ng/mL and LODs of 3.38 and 4.87 fg/mL for PSA and AFP, respectively.

Three different Raman tags were attached to Au nanoparticles which were then appropriately modified for the coupling of antibodies against three liver cancer markers, namely, AFP, CEA, and ferritin [140]. The respective capture antibodies were immobilized onto magnetic beads that facilitated the separation of immunocomplexes after the immunoreaction. The LODs achieved for AFP, CEA, and ferritin were 0.15, 20, and 4 pg/mL, respectively. 

Non-competitive immunoassays for the multiplexed detection of three cytokines, tumor necrosis factor-α (TNF-α), interferon-γ (IFN-γ), and interleukin-10 (IL-10), which are involved in cancer pathogenesis have also been developed using as labels Au nanoparticles that were firstly modified with Raman tags, covered with an Ag layer, and then the specific for each analyte antibodies were coupled [141]. These nanoparticles were combined with magnetic particles also modified with analyte-specific antibodies to allow the detection of TNF-α at concentrations as low as 4.5 pg/mL and up to 10 ng/mL. For the other two cytokines, analytical data are not provided, although they have been detected in a cell culture medium after providing appropriate stimuli. A SERS-microfluidic droplet platform has been used for the simultaneous detection of two other cytokines, the vascular endothelial growth factor (VEGF) and interleukin-8 (IL-8), secreted by a single cell [142]. For this purpose, water-in-oil droplets containing individual cells were mixed with Ag nanoparticles modified with antibodies against the two analytes and magnetic beads also modified with antibodies and two different Raman tags. The formation of immunocomplexes “turn on” the SERS signal of the Raman tags on the surface of magnetic nanoparticles due to the vicinity of the Ag nanoparticles. Following this approach, a LOD of 1.0 fg/mL and a dynamic range that extended up to 10 pg/mL were achieved for both cytokines.

Squamous cell carcinoma antigen (SCCA) and osteopontin, two markers of cervical cancer, have been detected using Au-Ag nanoshuttles as SERS labels and hydrophobic filter paper decorated with Au nanoflowers both modified with specific antibodies for the non-competitive immunodetection of these two markers [143]. The LODs obtained following this method were 8.628 pg/mL and 4.388 pg/mL for SCCA and osteopontin, respectively. The method was applied to detect the two markers in serum samples from healthy subjects and patients with cervical intraepithelial neoplasia I, II, and III, demonstrating the applicability of the method as a cervical cancer screening tool. Two markers of cervical cancer, SCCA and CA125, were also the target molecules in another report regarding the development of a SERS-based lateral flow immunoassay [144]. The labels used were polydopamine nanospheres decorated with Ag nanoparticles, Raman tags, and analyte-specific antibodies. The capture antibodies were spotted on a nitrocellulose membrane, and the formation of immunocomplexes was verified both by visual inspection and with Raman measurements. The LODs determined were 8.093 pg/mL for SCCA and 7.370 pg/mL for CA125 in human serum, and the assay’s dynamic range was from 10 pg/mL to 10 μg/mL. The simultaneous detection of SCCA and survivin, which are also markers of cervical cancer, has been reported [145]. A substrate with arrays of Au–Ag nanoboxes was modified with the respective capture antibodies, while Au–Ag nanoshells modified with Raman tags and antibodies were used as labels. LODs of 6 pg/m for SCCA and 5 pg/mL for survivin were achieved with a linear dynamic range for both analytes from 10 pg/mL to 10 μg/mL. Finally, the simultaneous determination of SCCA and CEA, also targeting cervical cancer, was performed in a microfluidic chip consisting of six functional units with a pump-free design that enabled parallel detection of multiple samples on an array of SiO_2_ particles decorated with Au nanoparticles and modified with the respective antibodies in combination with Ag nanocubes labeled with Raman tags and modified with analyte-specific antibodies [146]. The LODs of SCCA and CEA achieved were 0.45 pg/mL and 0.36 pg/mL, respectively, with a dynamic range from 1 pg/mL to 1 μg/mL.

After optimizing the distance between a substrate comprising 2D arrays of Au core-Ag shell nanoparticles and MBA-labeled Au nanoparticles as SERS labels, the optimum materials were employed to detect PSA, CEA, and CA19-9 at ranges from 1 pg/mL to 1 ng/nmL for PSA and CEA, and 10–40 U/mL for CA19-9 [147].

Four cancer biomarkers, namely PSA, AFP, CEA, and NSE, have been detected on substrates where the different antibodies have been immobilized sequentially on areas (1 mm × 1 mm squares) revealed by photolithography [148]. Au nanoparticles labeled with 4-MBA and antibodies against the four markers were used as labels in non-competitive assays that could detect all analytes at concentrations as low as 1 ng/mL. 

A gold microelectrode array fabricated by electrodeposition of Au to the bottom of numerous individual silica cavities was used as a substrate for the detection of AFP and CEA both by electrochemical and SERS measurements [149]. The detection antibodies were immobilized onto Au nanoparticles modified with oligonucleotide sequences to achieve signal enhancement by hybridization chain reaction. The sensor was able to detect 0.6 and 0.3 pg/mL of CEA and AFP, respectively, when SERS detection was employed.

A SERS immunochemical method for the diagnosis and prognosis of B cell hematological malignancies was developed based on the simultaneous detection of two surface markers (i.e., CD19 and CD20) in Raji cell lines as well as in clinical blood samples [150]. The assay implemented magnetic beads modified with an antibody against CD45 as the capture substrate for the leukocytes, while Ag nanoparticles modified with antibodies against CD19 or CD20 and labeled with Raman tags (4-MBA or DNTB) were used for the detection. The LOD of Raji cells achieved was five cells in five million K562 cells. The performance of the method was compared to that of flow cytometry by analyzing peripheral blood samples of both B cell hematological malignancy patients and samples from healthy individuals. It was shown that the SERS-based method provided a lower detection limit and reduced false negatives compared to flow cytometry. 

In Table S7, data concerning the multiplexed detection of different cancer markers by SERS biosensors are summarized. A comparison of multi-analyte SERS-based immunoassays with single-analyte ones in terms of detection sensitivity demonstrates the great potential of the SERS technique for the simultaneous determination of multiple biomarkers in human biological samples. This feature is particularly important for the diagnosis and follow-up of cancer since rarely the monitoring of a single biomarker levels in serum provides sufficient evidence.

## 5. Conclusions and Outlook

Surface-enhanced Raman spectroscopy has emerged as a powerful analytical strategy for immunochemical biomarker detection. This is evident from the many reports regarding the immunochemical detection of biomarkers related to cancer diagnosis and prognosis that have been presented in detail in this review article. 

A quick glance at all these reports shows that all the assays developed are characterized by high detection sensitivity that, in some cases, surpasses that of standard microtiter well enzyme immunoassays. The high detection sensitivity allows for the targeted analyte detection at very low concentrations, usually several orders of magnitude lower than those encountered in biological fluids. This means that in practice, the biological samples can be diluted several times prior to their analysis, thus minimizing effects from the matrix and facilitating the method’s clinical application. In addition, although not all these reports evaluate the developed SERS sensors with respect to their selectivity, wherever these data are available, they show very high selectivity towards the targeted analyte with respect to interfering substances (e.g., other cancer markers or serum proteins). This high selectivity does not characterize only the immunosensors discussed in this review for which the selectivity arises from the selective binding properties of the antibodies used, but also sensors employing other binding moieties, e.g., molecularly imprinted polymers.

Another feature that promotes SERS application in the field of cancer biomarker detection is the potential application at the point of need through the development of microfluidic devices or lateral flow devices. These devices incorporate sample processing, require much fewer reagents than the standard immunoassays, and in most cases, increase the reaction speed allowing for fast but equally accurate results.

Another very interesting feature of SERS-based immunosensors is their multiplexing capability. To this end, two approaches have been exploited, the spatial separation of the solid-phase reagents (i.e., antigens or antibodies) by spotting them onto discrete positions of the same substrate in a similar way to that microarrays are realized, or the implementation of different labels, one for each analyte, the signals of which could be distinguished when all of them are present in a mixture. The first approach is easier to realize since the different reaction sites are well separated, and theoretically, a very high number of different molecules can be detected in a single run. The practical difficulty lies, as in the case of antigen or antibody microarrays that implement other types of detection (e.g., fluorescent labels), in the harmonization of assays that should be run simultaneously, i.e., the definition of assay conditions that achieve the required for the application detection sensitivity and dynamic range for all assays. The second approach for multiplexed determination requires the existence of labels whose Raman signals are easily distinguished from each other. Thus, the number of biomarkers that can be detected simultaneously is usually limited to two or three analytes. In this case, false-positive results might arise from cross-talk between the different labels. A way to avoid these effects is the dual labeling of both the detection molecules and the substrate; however, that adds to the complexity of the assay and signal interpretation.

Nonetheless, as more new materials are exploited both as substrates and tags for SERS-based biosensing, the above-mentioned limitations might be surpassed in the near future. For example, most of the reports regarding cancer biomarkers detection by SERS reviewed here employ substrates or tags made of Au or Ag. There is, however, ongoing research on nanostructures made of other metals such as Zn or Cu, and of course, there is the possibility of using metal alloys. In addition to different materials, new shapes and configurations of nanoparticulate materials used as substrates or labels might improve the analytical performance of multiplexed SERS-based immunoassays. Thus, the future research focus with respect to labels will be to find materials and shapes that could provide not only signal amplification but also signal stability. Regarding labels, the combination of an inorganic core with a metal cell is the approach more widely used already and possibly the one more popular in the future.

Another aspect that cannot be overlooked is the development of portable Raman spectroscopes that would be combined with automated microfluidic or lateral flow devices to provide fast determinations at the point of need, preferably at a cost competitive to that of already existing methods. A small compromise regarding the detection sensitivity might be acceptable with respect to that achieved using laboratory-based instruments.

Although the subject of this review is to present the advancements in the field of immunosensors for cancer biomarkers detection, the implementation of other binding moieties, such as aptamers and molecularly imprinted polymers, should not be excluded since they have some advantages in terms of chemical and storage stability but their application for detection of targeted molecules to complex matrices such are the biological samples are still not fully evaluated.

Thus, despite the impressive advancements described in this article and the excellent analytical characteristics of the so-far developed SERS-based immunosensors, there is a lot of room for improvement and future innovation regarding the development of SERS-based methods for cancer biomarkers detection so as these methods to compete and/or replace at some point the ones currently used in everyday clinical practice.

## Data Availability

No new data were created or analyzed in this study. Data sharing is not applicable to this article.

## Supplementary Materials

The following supporting information can be downloaded at: https://www.mdpi.com/article/10.3390/ma16103733/s1, Table S1: SERS-based detection of PSA; Table S2: SERS-based detection of AFP; Table S3: SERS-based detection of CEA; Table S4: SERS-based detection of cancer markers MUC4 and HE4; Table S5: SERS-based detection of different cancer markers; Table S6: SERS-based detection of interleukins as cancer markers; Table S7: Multiplexed SERS-based detection of cancer markers.
