# Supplementary material for: SERS Immunosensors for Cancer Markers Detection
Source: Materials (Basel). 2023 May 15;16(10):3733. doi: 10.3390/ma16103733 (PMC10221005; doi:10.3390/ma16103733)
Supplement: Supplementary file 1 [file materials-16-03733-s001.zip › materials-2363542-supplementary.pdf]

SUPPLEMENTARY MATERIAL

SERS Immunosensors for Cancer Markers Detection

Georgia Geka[1], Anastasia Kanioura[1], Vlassis Likodimos[2], Spiros Gardelis[2], Nikolaos Papanikolaou[3], Sotirios Kakabakos[1] and Panagiota Petrou[1,*]

[1] Immunoassays/Immunosensors Lab, Institute of Nuclear & Radiological Sciences & Technology, Energy & Safety, NCSR "Demokritos", 15341 Aghia Paraskevi, Greece

[2] Section of Condensed Matter Physics, Department of Physics, National and Kapodistrian University of Athens, 15771 Zografou, Greece

[3] Institute of Nanoscience & Nanotechnology, NCSR "Demokritos", 15341 Aghia Paraskevi, Greece

Table S1. SERS-based detection of PSA.

Biomarker	Disease	Sample	Substrate/Label	LOD	Working range	Ref.
PSA	prostate cancer	blood serum	microtiter plate/Au nanoparticles modified with catalase + aggregated Au nanoparticles labeled with 4-MBA	10^{-9} ng/nL	10^{-9}-10^{-7} ng/mL	[61]
		buffer	quartz slide/silica-coated Ag nanorods modified with 4-MBA	0.3 fg/mL	0.3 fg/mL-30 μg/mL	[62]
		synthetic human serum	magnetic beads /Au nanoparticles labeled with malachite green isothiocyanate	0.1 ng/mL	0.1-200 ng/mL	[63]
		blood serum	magnetic beads/Au nanoparticles modified with malachite green isothiocyanate	0.01 ng/mL	0.01-100 ng/mL	[64]
		blood serum	Ag nanoparticles on graphene oxide/ streptavidin-glucose oxidase	0.23 pg/mL	0.5-5.00 pg/mL	[65]
		blood serum	Au nanowires on silica wafer/ Ag aggregates modified with 2-naphthalenethiol	1 fg/mL	1 fg/mL-1 μg/mL	[66]

	blood serum	polyacrylamide gel containing a zinc finger peptide/magnetic Au nanoparticles/ ZnO-Au nanocomplexes	0.65 pg/mL	1 pg/mL-10 ng/mL	[67]
	blood serum	microparticles with Fe_3O_4 core coated with TiO_2 and Au nanoparticles (Fe_3O_4@TiO_2@Ag microparticles)	16.25 pg/mL	0.1 ng/mL-100 μg/mL	[68]
	blood serum	SiC sandpaper sputtered with Ag/ microparticles with Fe_3O_4 core coated with TiO_2 and Au nanoparticles modified with 4-MBA	1.871 pg/mL	1.871 pg/mL-100 μg/mL	[69]
	buffer	electrospun polycaprolactone fibers decorated with Ag nanoparticles/Au nanoparticles modified with 4-MBA	1 pg/mL	1 pg/mL-1 μg/mL	[70]
	blood serum	magnetic nanoparticles/Au nanoparticles modified with 4,4′-dipyridyl	5.0 pg/mL	5.0-500 pg/mL	[71]
	blood serum	magnetic molecularly imprinted polymers prepared using tannic acid as monomer and diethylenetriamine as cross-linker/Au modified with 5,5′ -dithiobis-(2-nitrobenzoic acid)	0.9 pg/mL	3.2 pg/mL-1 μg/mL	[72]
free-PSA	blood serum	Au slide/nanoparticles with Au core modified with 1,4-benzenedithiol and covered by Au layer	2.0 pg/mL	10 pg/mL-10 ng/mL	[73]

Table S2. SERS-based detection of AFP.

Biomarker	Disease	Sample	Substrate/Label	LOD	Working range	Ref.
AFP	liver cancer	buffer	Au array/hollow Au nanospheres	1 ng/mL	1-10 ng/mL	[75]
		buffer	glass slide with Au nanoparticles/Au nanoparticles modified with 4-MBA	100 pg/mL	1-100 ng/mL	[76]
		buffer	$NiCo_2O_4$ nanorods decorated Ag nanoparticles/SiO_2 microspheres with Ag nanoparticles and 4-MBA	2.1 fg/mL	2.1 fg/mL-21 ng/mL	[77]
		blood serum	nitrocellulose membrane/Au nanospheres coated with an Ag layer, an ultrathin silica shell, and Au nanosphere satellites	0.3 fg/mL	1 fg/mL-1 ng/mL	[78]
		blood serum	nitrocellulose membrane/Au-Ag nanostars modified with 4-MBA and covered with a SiO2 layer	0.72 pg/mL	3 pg/mL-3 μg/mL	[79]
		blood serum	MoS_2 sheets/rhodamine 6G/ Ag-coated Au nanocubes	0.03 pg/mL	1 pg/mL-10 ng/mL.	[80]
AFP		blood serum	molecular imprinted polymer on a glass slide/Ag nanoparticles modified with boronic acid	1 ng/mL	1 ng/mL-10 μg/mL	[81]
AFP<br>AFP-L3		blood serum	molecular imprinted polymers spotted onto Au nanoparticles-coated glass slide/Ag nanoparticles labelled with Raman reporters and covered with molecular imprinted molecules	0.1 ng/mL<br>0.1 ng/mL	0.1 ng/mL-10 μg/mL<br>0.1-8 ng/mL	[82]
AFP-L3		buffer	Ag/Fe/Ag films coated polystyrene colloidal particles monolayer modified with 5,5'-	0.5 ng/mL	0.5-8 ng/mL	[83]

		dithiobis(succinimidyl-2-nitrobenzoic acid			
AFP<br>AFP-L3	blood serum	silicon substate with Ag nanoparticles modified with 4-MBA/Au nanoparticles modified with 5,5-dithiobis(succinimidyl-2-nitrobenzoate	0.5 ng/mL<br>8 ng/mL	0.5-1000 ng/mL<br>8-1000 ng/mL	[84]

Table S3. SERS-based detection of CEA.

Biomarker	Disease	Sample	Substrate/Label	LOD	Working range	Ref.
CEA	lung cancer	buffer	silica-coated magnetic microspheres/ hollow gold nanospheres modified with 4-MBA	10 pg/mL	up to 100 ng/mL	[86]
	n.d.	buffer	magnetic nickel–iron core particles with Au shell/Au nanoparticles modified with 4-MBA	0.1 ng/mL	0.1-10 ng/mL	[87]
	colorectal cancer	blood serum	Fe_2O_3@Au nanoparticles/Au nanoparticles modified with 4-MBA	0.1 ng/mL	1-50 ng/mL	[88]
	colorectal cancer	whole blood	magnetic nanoparticles/Au nanoparticles labeled with 4,4′-bipyridine	1 pg/mL	1 pg/mL-1 μg/mL	[89]
			glass carbon electrodes with Au nanoparticles and chitosan/Au nanoparticles modified with polydopamine and decorated with Nile blue	1.38 ng/mL	2 -100 ng/mL	[90]
	different cancer types	blood plasma	Fe_3O_4@Au nanoparticles incorporated onto delaminated $Ti_3C_2T_x$ MXene sheets/ MoS_2 nanoflowers@Au nanoparticles modified with 4-MBA	0.033 pg/mL	0.0001-100 ng/mL	[91]
	n.d.	blood serum	polymer made of dopamine, m-aminophenylboronic acid monohydrate, and ammonium persulfate embedded Au nanoparticles modified with 4-mercaptobenzonitrile & Ag aggregates/ Au nanoparticles modified with ethynylbenzene & covered by poly-dopamine	0.064 pg/mL	0.1 pg/mL- 10 μg/mL	[92]
	colon cancer	blood serum	molecularly imprinted polymer of APTES, UPTES), IBTES, and	100 pg/mL	1 ng/mL–10 μg/mL	[93]

		TEOS, over Au nanoparticles/ molecularly imprinted polymer of dopamine covered Ag nanoparticles			
liver cancer	blood serum	boronate-affinity molecularly imprinted polymer prepared using 4-vinylbenzeneboronic acid as monomer and ethylene glycol dimethacrylate as cross-linked/Au nanoparticles labeled 4-mercaptophenylboronic acid	0.1 ng/mL	0.1 ng/mL-1 mg/mL	[94]
n.d.	buffer	molecularly-imprinted polymer of gallic acid on Au-screen-printed electrodes/Au nanostars coupled to 4-aminothiophenol	1.0 ng/mL	1.0 -1000 ng/mL	[95]

Table S4. SERS-based detection of cancer markers MUC4 and HE4.

Biomarker	Disease	Sample	Substrate/Label	LOD	Working range	Ref.
MUC4/ Ca19-9	pancreatic cancer	cell lysate/ blood serum	silicon wafers with Au layer/Au nanoparticles modified with 4-nitrobenzenethiol	33 ng/mL/ 0.8 U/mL	100 ng/mL-10 μg/mL/ 0.8 U/mL-12 U/mL	[101]
MUC4		blood serum	atomically smooth mica with Au deposition/Au nanoparticles decorated with 4-nitrobenzenethiol	n.d.	n.d.	[102]
		buffer	silicon substrate with Au nanoflowers/Au nanoflowers modified with 4-MBA	0.1 ng/mL	0.1 ng/mL-10 μg/mL	[103]
HE4		buffer	Fe_3O_4@Au nanoparticles/Au nanoparticles modified with 4-MBA	100 fg/mL	1 pg/mL-10 ng/mL	[107]
		buffer	single-crystalline Au nanoplate/Au nanoparticles modified with malachite green isothiocyanate	0.31 fg/mL	0.31 fg/mL-31 ng/mL	[108]

*n.d. = not defined

Table S5. SERS-based detection of different cancer markers.

Biomarker	Disease	Sample	Substrate/Label	LOD	Working range	Ref.
VEGF	different cancer types	blood serum	Au triangle nanoarray/Au nanostars modified with malachite green isothiocyanate	7 fg/mL	0.1 pg/mL-10 ng/mL	[109]
HER2	breast cancer	blood serum	Au electrodes incorporated onto a microfluidic device/Au@Ag nanoshells modified with malachite green isothiocyanate or fluorescent tags	10 fg/mL	1 fg/mL-100 pg/mL	[110]
CA19-9	colorectal/ pancreatic cancer	blood serum	F_3O_4/TiO_2/Au nanoparticles	$5.65x10^{-4}$ IU/mL	0.001-1000 IU/mL	[111]
p53 p53R175H	different cancer types	blood serum	silicon modified with amine-silane & glutaraldehyde/Au nanoparticles with diazonium groups	10^{-17} M	10^{-17}-10^{-10} M	[112]
squamous cell carcinoma	cervical cancer	peripheral blood	polydopamine resin microspheres coated with Au nanoparticles/ hollow Au nanocages modified with 4-MBA	8.03 pg/mL	10^{-5}-10^{-10} M	[113]
B7 homolog 6 protein	cervical cancer	blood serum	Au coated silicon substrates modified with zwitterionic L-cysteine/Au nanoparticles functionalized with ATP	10.8 fg/mL	n.d.	[114]
Human carboxylesterase 1	hepatocellular carcinoma	blood serum	raspberry-like Fe_3O_4/SiO_2/Ag nanocomposites/Ag nanoparticles modified with 4-MBA	0.1 ng/mL	0.1 ng/mL-1.0 mg/mL	[115]

galectin-3-binding protein (LGALS3BP; 90K)	different cancer types	blood serum	Silicate glass slides coated with thin gold layer	15 ng/mL	n.d.	[116]
Ferritin	liver cancer	buffer	sandpaper modified with Ag nanoparticles/mesoporous hybrid SiO_2 particles coated with Au nanoparticles and modified with 4-MBA	31.6 fg/mL	1 pg/mL-10 μg/mL	[117]
extracellular vesicles	different cancer types	buffer	Fe_3O_4 nanoparticles with a silica shell/nanorods with Au core and Ag shell modified with 5,5′-dithiobis(2-nitrobenzoic acid)	1200 exosomes	n.d.	[118]
extracellular vesicles	different cancer types	plasma	magnetic beads/ Au nanoparticles modified with different Raman tags	2.3×10^6 particles/mL	2.3×10^6-$2.3\text{x}10^8$ particles/mL	[119]
α-thrombin<br>tumor necrosis factor-α	different cancer types	blood serum	Au film with methylene blue/ Au nanoparticles labeled with 4-nitrobenzenethiol	86 pM<br>0.07 nM	86 pM-1 nM<br>0.07 nM-1.2 nM	[120]
BRCA1 protein	breast cancer	blood serum	Ag grains	0.1 ng/mL	n.d.	[121]

*n.d.=not determined

Table S6. SERS-based detection of interleukins as cancer markers.

Biomarker	Disease	Sample	Substrate/Label	LOD	Working range	Ref.
IL-6	different cancer types	buffer	glass slide/Au-Ag nanoshells stabilized with a self-assembled monolayer of 5,5′-dithiobis(2-nitrobenzoic acid) molecules comprising terminal mono- and tri-ethylene glycol	1 pg/mL	1 pg/mL-1 µg/mL	[123]
IL-8	gastric & breast cancer	human serum	Au nanoparticles/Au nanocages modified with 4-MBA	6.04 pg/mL	10 pg/mL-1 µg/mL	[124]
IL-6<br>IL-8<br>IL-18	different cancer types	blood plasma	Ag-Au substate incorporated into microfluidic device/Au nanoparticles modified with 5,5′-dithio-bis(2-nitro-benzoic acid), fuchsin or 4-MBA	3.8 pg/mL<br>7.5 pg/mL<br>5.2 pg/mL	3.8 pg/mL-30 ng/mL<br>7.5 pg/mL-30 ng/mL<br>5.2 pg/mL-30 ng/mL	[125]

Table S7. Multiplexed SERS-based detection of cancer markers.

Biomarker	Disease	Sample	Substrate/Label	LOD	Working range	Ref.
AFP<br>angiotensin	different cancer types	buffer	micropatterned Au film/hollow Au nanospheres	0.1 pg/mL<br>1.0 pg/mL	0.1 pg/mL-0.1 μg/mL<br>1.0 pg/mL-0.1 μg/mL	[125]
CEA<br>AFP	lung cancer	serum	magnetic beads/hollow Au nanospheres with Raman tags	n.d.<br>n.d.	up to 100 ng/mL	[126]
MMP-7<br>CA19-9	pancreatic cancer	serum	Au coated slide/Au nanoparticles modified with 5,5′-dithiobis(succinimidyl-2-nitrobenzoate)	2.28 pg/mL<br>34.5 pg/mL	2.28 pg/mL-6 ng/mL<br>34.5 pg/mL-18 ng/mL	[127]
CA125<br>HER2<br>HE4<br>eotaxin-1	breast cancer	serum	Au nanostars/Au nanostars modified with Rhodamine 6G	15 fM<br>17 fM<br>21 fM<br>6.5 fM	15 fM-10 pM<br>17 fM-10 pM<br>21 fM-10 pM<br>6.5 fM-10pM	[128]
CA15-3<br>CA27-29<br>CEA	breast cancer	serum	quartz slide using/Au nanostars modified with 4-nitrothiophenol embedded in SiO_2	0.99 U/mL<br>0.13 U/mL<br>0.05 ng/mL	0.1 U/mL-500 U/mL<br>0.1 U/mL-500 U/mL<br>0.1 ng/mL-500 ng/mL	[129]
CEA<br>AFP	different cancer types	serum	Ag nanoparticles/periodically arranged monodisperse nanoparticles of SiO_2 decorated with Ag particles	$6.6 x10^{-6}$ ng/mL<br>$7.2x10^{-5}$ ng/mL	0.01 pg/mL-1000 ng/mL<br>0.1 pg/mL-1000 ng/mL	[130]
CEA<br>cytokeratin-19	lung cancer	serum	electrodes modified with chitosan stabilized Au nanoparticles/aminosalicylic acid-based resin microspheres modified Raman tags and Au nanoparticles	0.01 ng/mL<br>0.04 ng/mL	0.05-80 ng/mL	[131]
CEA<br>AFP<br>CA125		serum	superparamagnetic Fe_3O_4@SiO_2 particles/hybrid multilayered nanoshells	0.1 pg/mL<br>n.d.<br>n.d	0.1 pg/mL-1 ng/mL<br>n.d.<br>n.d.	[132]

			prepared by assembly of small Ag nanoparticles at the surface of silica particles modified with Raman tags			
CEA<br>neuron-specific enolase	lung cancer	serum	magnetic nanoparticles/flower-like gold nanoparticles with Raman tags	1.48 pg/mL<br>2.04 pg/mL	1 fg/mL-1 ng/mL	[133]
PSA<br>AFP<br>CA19-9	different cancer types	serum	SiC sandpaper with sputtered Ag film/Si nanoparticles coated with SiO_2	1.79 fg/mL<br>0.46 fg/mL<br>1.3×10^{-3}U/mL	1.79 fg/mL-71.87 ng/mL<br>0.46 fg/mL-95.75 ng/ml<br>1.3×10^{-3}-10^{3} U mL−1	[134]
PSA<br>prostate-specific membrane antigen<br>human kallikrein 2	prostate cancer	serum	SiC sandpaper with a sputtered Ag film/<br>Ag nanoparticles with 4-MBA	0.46 fg/mL<br>1.05 fg/mL<br>0.67 fg/mL	0.46 fg/mL-1 ng/mL<br>1.05 fg/mL-1 ng/mL<br>0.67 fg/mL-1 ng/mL	[135]
CEA<br>AFP	different cancer types	serum	Au nanohoneycomb arrays/Au nanostars modified with 4-MBA or DTNB	0.44 ng/ml<br>0.40 ng/ml	0.5-100 ng/mL<br>0.5-100 ng/mL	[136]
CA153<br>CA125<br>CEA	different cancer types	serum	polydimethylosiloxame which Ag nanoparticles/Ag aggregates labelled with Raman tags	0.01 U/mL<br>0.01 U/mL<br>1pg /mL	0.01-1000 U/mL<br>0.01-1000 U/mL<br>1 pg/mL-100 ng/mL	[137]
PSA<br>AFP	different cancer types	serum	Au-film hemisphere array/silica beads coated with Ag nanoparticles and modified with 4MBA or 4-nitrothiophenol	3.38 fg/mL<br>4.87 fg/mL	10 fg/mL-400 ng/mL<br>10 fg/mL-400 ng/mL	[138]
AFP<br>CEA<br>FER	liver cancer	serum	magnetic beads/Au nanoparticles modified with Raman tags	0.15 pg/mL<br>20 pg/mL<br>4 pg/mL	0.5–500 pg/mL<br>50–2000 pg/mL<br>10–200 pg/mL	[139]
TNF-α	different cancer	cell	magnetic particles/Au	4.5 pg/mL<br>n.d.	4.5 pg/mL-10 ng/mL<br>n.d.	[140]

interferon-γ<br>interleukin-10	types	culture medium	nanoparticles modified with Rama tags and covered with an Ag layer	n.d.	n.d.	
VEGF<br>interleukin-8	different cancer types	cell culture medium	Ag nanoparticles/magnetic beads modified with Raman tags	1.0 fg/mL<br>1.0 fg/mL	1.0 fg/mL-10 pg/mL	[141]
SCCA<br>osteopontin	cervical cancer	serum	hydrophobic filter paper decorated with Au nanoflowers/Au-Ag nanoshuttles	8.628 pg/mL<br>4.388 pg/mL	10 pg/mL-10 μg/mL	[142]
SCCA<br>CA125	cervical cancer	serum	nitrocellulose membrane/ polydopamine nanospheres decorated with Ag nano-particles and Raman tags	8.093 pg/mL<br>7.370 pg/mL	10 pg/mL to 10 μg/mL.	[143]
SCCA<br>Surviving	cervical cancer	serum	arrays of Au–Ag nanoboxes/Au–Ag nanoshells modified with Raman tags	6 pg/mL<br>5 pg/mL	10 pg/mL-10 μg/mL<br>10 pg/mL-10 μg/mL	[144]
SCCA<br>CEA	cervical cancer	serum	microfluidic chip consisting of six detection areas of SiO_2 particles decorated with Au nanoparticles/Ag nanocubes labeled with Raman tags	0.45 pg/mL<br>0.36 pg/mL	1 pg/mL-1 μg/mL<br>1 pg/mL-1 μg/mL	[145]
PSA<br>CEA<br>CA19-9	different cancer types	buffer	2D arrays of Au core-Ag shell nanoparticles/4-MBA-labeled Au nanoparticles	1 pg/mL<br>1 pg/mL<br>10 U/mL	1 pg/mL-1 ng/mL<br>1 pg/mL-1 ng/mL<br>10-40 U/mL	[146]
PSA<br>AFP<br>CEA<br>NSE	different cancer types	buffer	photopatterned substrate/Au nanoparticles labeled with 4-MBA	0.19 ng/mL<br>0.60 ng/mL<br>0.13 ng/mL<br>0.26 ng/mL	0.5-50.0 ng/mL<br>1.0–100.0 ng/mL<br>0.5–50.0 ng/mL<br>1.0–100.0 ng/mL	[147]
CEA<br>AFP	different cancer types	serum	gold microelectrode array with electrodeposited	0.6 pg/mL<br>0.3 pg/mL	5-200 pg/mL<br>2-100 pg/mL	[148]

			Au/Au nanoparticles			
CD19 CD20 in Raji cell lines	B cell hematological malignancies	peripheral blood	magnetic beads/Ag nanoparticles labelled with Raman reporters (4-MBA or DNTB)	5 cells/mL in 5×10^6 total cells	5-5000 cells/mL in 5×10^6 total cells	[149]

*n.d. = not defined
